# Trends in Antibiotic-Resistant Bacteria Isolated from Screening Clinical Samples in a Tertiary Care Hospital over the 2018–2022 Period

**DOI:** 10.3390/antibiotics12081314

**Published:** 2023-08-14

**Authors:** Delphine Lemonnier, Marine Machuel, Odile Obin, Gaëtan Outurquin, Crespin Adjidé, Catherine Mullié

**Affiliations:** 1 Unité de Prévention du Risque Infectieux, Centre Hospitalier Universitaire Amiens-Picardie, 80054 Amiens, France; lemonnier.delphine@chu-amiens.fr; 2 Laboratoire Hygiène Risque Biologique & Environnement, Centre Hospitalier Universitaire Amiens-Picardie, 80054 Amiens, Francecrespin.adjide@chu-amiens.fr (C.A.); 3 Laboratoire AGIR UR UPJV 4294, UFR de Pharmacie, Université de Picardie Jules Verne, 80037 Amiens, France

**Keywords:** multidrug-resistant bacteria, screening, carbapenamase-producing enterobacteria, extended-spectrum beta-lactamase-producing enterobacteria, methicillin-resistant *Staphylococcus aureus*, vancomycin-resistant enterococci

## Abstract

To assess the putative impact of the COVID-19 pandemic on multidrug-resistant (MDR) bacteria recovered from routine screening samples and, more globally, the trends in time to first positive screening sample and carriage duration of those bacteria in patients admitted to a tertiary hospital, data from laboratory results were retrospectively mined over the 2018–2022 period. No significant differences could be found in the number of positive patients or MDR isolates per year, time to positive screening, or carriage duration. Extended-spectrum beta-lactamase producers were dominant throughout the studied period but their relative proportion decreased over time as well as that of meticillin-resistant *Staphylococcus aureus*. Meanwhile, carbapenemase-producing enterobacteria (CPE) proportion increased. Among the 212 CPE isolates, *Klebsiella pneumoniae* and *Escherichia coli* were the more frequent species but, beginning in 2020, a significant rise in *Enterobacter cloacae* complex and *Citrobacter freundii* occurred. OXA48 was identified as the leading carbapenemase and, in 2020, a peak in VIM-producing enterobacteria linked to an outbreak of *E. cloacae* complex during the COVID-19 pandemic was singled out. Finally, a worrisome rise in isolates producing multiple carbapenemases (NDM/VIM and mostly NDM/OXA48) was highlighted, especially in 2022, which could lead to therapeutic dead-ends if their dissemination is not controlled.

## 1. Introduction

With a projected 10 million yearly deaths attributed to antimicrobial resistance in 2050, multidrug-resistant (MDR) bacteria are now widely recognized as a major health threat throughout the globe, with the World Health Organization (WHO) urging for the search for new antibiotic treatments to tackle infections driven by those bacteria [1,2,3]. Colonization of patients with MDR bacteria has been pointed out as an important risk factor for the development of infections caused by these microorganisms [4,5,6] and healthcare settings identified as a major place for their acquisition and dissemination [7,8]. Therefore, several national entities have established guidelines and/or recommendations to limit the acquisition and spread of MDR bacteria in healthcare settings [9,10,11,12,13]. Most of these infection prevention and control (IPC) measures include the screening of high-risk patients and/or of patients admitted to high-risk wards. This high-risk classification is based on various parameters encompassing the patient’s clinical conditions (mostly including immunocompromized individuals such as oncologic patients), individuals returning from travel abroad, previously hospitalized patients, individuals with multiple risks as well as patients admitted in an intensive care unit (ICU) [7,8]. In the shortterm, these surveillance schemes allow for the rapid identification of MDR carriers and subsequent implementation of adapted IPCs in healthcare settings. From a long-term point of view, these screenings also provide vital epidemiological information on the patterns of spread, carriage prevalence, and carriage duration within healthcare settings but also on which MDR bacteria could now be imported from the community rather than acquired during patients’ stay in those settings. In this work, the screening surveillance data of a French tertiary hospital were mined over the 2018–2022 period to determine which MDR bacteria were the most frequently identified and whether (i) most MDR bacteria were community- or hospital-acquired, (ii) the time to the first positive sampling was similar for the main bacterial species encountered, and (iii) the high-risk ward epidemiology was different from low-risk wards. We also aimed to uncover whether significant time variations occurred over the studied period, knowing that it included the COVID-19 pandemic, which has been shown to impact the transmission of MDR bacteria in healthcare settings [14].

## 2. Results

### 2.1. Number of Positive Patients, Isolates, Time to First Positive Screening, and Length of Carriage

A total of 2442 patients were included over the 5 years, yielding an average of 488.4 ± 61.55 positive patients per year. The details of yearly values for the number of patients as well as the time to first positive screening sample and length of MDR carriage are presented in Table 1.

The distribution of the number of patients carrying MDR bacteria did not significantly vary over time as compared to an expected uniform distribution of patients throughout the five years (*p* = 0.975, Kolgromov–Smirnov test), with the highest proportion of MDR carriers found in 2019 and the lowest in 2021, respectively (Table 1). The time to the first positive screening sample and length of carriage did not significantly change over time either (Kruskal–Wallis test, *p* = 0.5629 and *p* = 0.7009, respectively).

### 2.2. Time-Dependent Variations in the Number of MDR Isolates, MDR, and Carbapenemase Types

**Figure 1:**
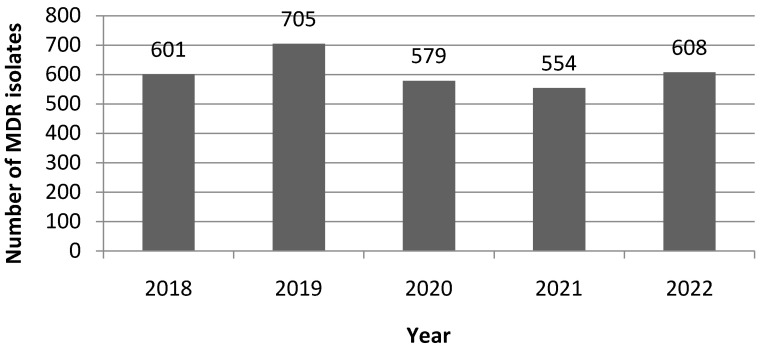
Yearly (first occurrences only for a given patient and for each year) numbers of MDR isolates.

Over the 2018–2022 period, when only the first occurrence of isolation for a given bacterium/patient couple is taken into account, the overall number of isolated MDR bacteria was 2842 (Table S1). The number of MDR per year is displayed in Figure 1.The observed distribution of MDR isolates was not statistically different from an expected homogenous distribution over the five years (*p* = 0.975, Kolgromov–Smirnov test). The highest number of MDR isolation was obtained in 2019 and the lowest in 2021 with 23.1% and 18.2% of the overall number of harvested MDR isolates, respectively.

**Figure 2:**
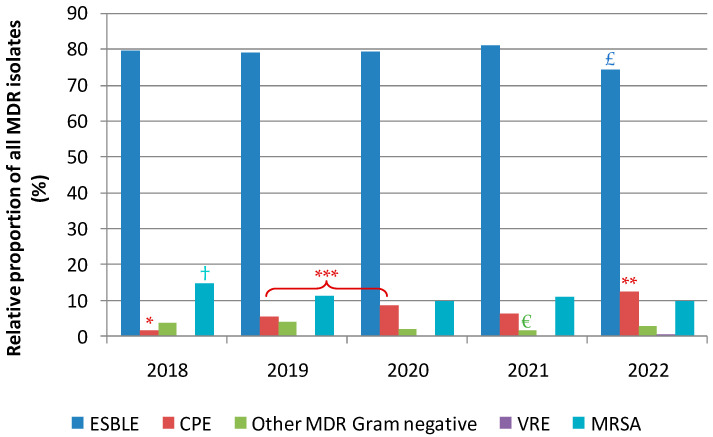
Yearly distribution of the main MDR categories. MRSA: Methicillin-resistant *Staphylococcus aureus*; VRE: Vancomycin-resistant enterococci; CPE: Carbapenem-producing enterobacteria; ESBLE: Extended-spectrum beta-lactamase-producing enterobacteria. ^£^: relative proportion for ESBLE significantly lower than those in all other years at *p* < 0.05 (Pearson’s Chi-square test); ^†^: relative proportion for MRSA significantly higher than those in years 2020, 2021, and 2022 at *p* < 0.05 (Pearson’s Chi-square test); *: relative proportion for CPE significantly lower than those in all other years at *p* ≤ 0.0005 (Pearson’s Chi-square test); **: relative proportion for CPE significantly higher than those in years 2019, 2020 and 2021 at *p* ≤ 0.04 (Pearson’s Chi-square test); ***: significant difference at *p* = 0.0293 (Pearson’s Chi-square test); ^€^: relative proportion for other MDR Gram-negative bacteria significantly lower than those in years 2018 and 2019 at *p* ≤ 0.04 (Pearson’s Chi-square test).

Globally, with 88.3% of the total isolates, Gram-negative MDR bacteria far outweighed Gram-positive ones. Extended-spectrum beta-lactamase-producing enterobacteria (ESBLE) were the most prevalent MDR bacteria traced down throughout the 5 years, with relative proportions ranging from 74.2 to 81% (Figure 2). However, their relative proportion among MDR isolates significantly decreased in 2022 as compared to the four previous years (Pearson’s Chi-square test, *p* < 0.05).

Similarly, methicillin-resistant *Staphylococcus aureus* (MRSA) strains registered a lasting and statistically significant decrease in their relative contribution to overall MDR isolates, going from 14.8% in 2018 to 9.9% in 2022 (Figure 2). An opposite trend was witnessed for carbapenemase-producing enterobacteria (CPE) with a significant rise in 2022 as compared to all other years, with their relative proportion among MDR isolates reaching 12.5% in 2022 (Figure 2).

Over the 5 years, 212 non-redundant carbapenemase-producing isolates were recovered. The identified carbapenemases were (by order of decreasing frequency): OXA48 (50.5%), NDM (21.7%), VIM (12.7%), OXA48/NDM combination (10.4%), KPC (2.4%), VIM/NDM combination (1.4%) and IMP (1%).

**Figure 3:**
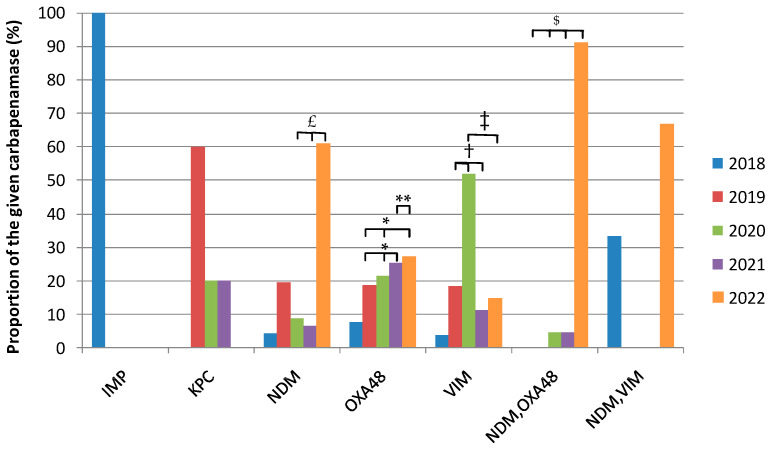
Yearly relative proportions in identified carbapenemases or carbapenemase combinations among carbapenemase-producing isolates. Significantly higher proportion for OXA48 (Pearson’s Chi-square test) at *: *p* < 0.05 for 2021 and 2022 compared to 2019 and 2020; ** *p* < 0.0001 for 2022 as compared to 2021. Significantly higher proportion for VIM (Pearson’s Chi-square test) at ^†^: *p* < 0.05 for 2020 compared to 2019 and 2021; ^‡^ *p* < 0.0001 for 2020 as compared to 2022. Significantly higher proportion for NDM/OXA48 (Pearson’s Chi-square test) at ^$^: *p* < 0.006 for 2022 compared to 2019, 2020, and 2021. Significantly higher proportion for NDM (Fisher’s exact test) at ^£^: *p* < 0.05 for 2022 as compared to 2020 and 2021.

As can be seen in Figure 3, a steady increase in the proportion of OXA48-carrying strains was found over the 5-year period with a significantly higher value in 2022 as compared to all other years, with the exception of 2018. Moreover, peaks in VIM- and NDM/OXA48-producing isolates were observed in 2020 and 2022, respectively. As very few isolates produced KPC, IMP, and the NDM/VIM combination, no significant differences could be witnessed over the 5 years.

### 2.3. Species Isolation and Resistance Frequencies

**Figure 4:**
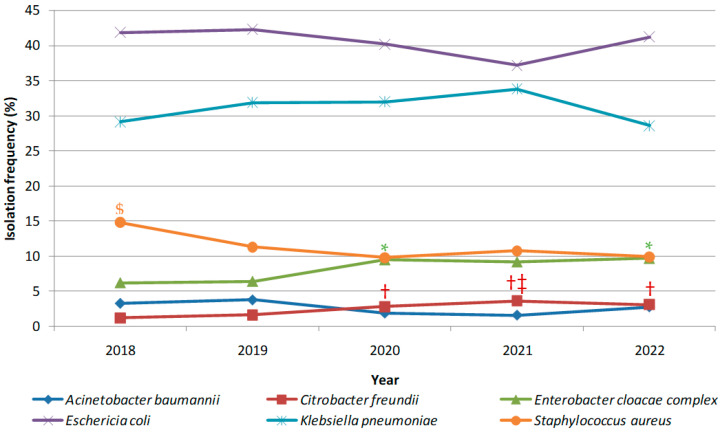
Isolation frequencies (expressed as percentages of the overall number of isolated MDR bacteria for a given year) of the main MDR species isolated. Relative proportion significantly higher at (Pearson’s Chi-square test): ^$^ *p* < 0.05 for MRSA compared to those in years 2020, 2021, and 2022; * *p* < 0.05 for *E. cloacae* complex compared to those in years 2018 and 2019; ^†^ *p* < 0.05 for *C. freundii* compared to that of 2018; ^‡^ *p* < 0.02 for *C. freundii* compared to that of 2019.

Isolation frequencies of the six most prevalent MDR species are shown in Figure 4. The two main contributors over the study period remained *Escherichia coli* and *Klebsiella pneumoniae*. No significant variation over time in their relative proportions among MDR isolates could be evidenced by the statistical analysis. The same finding held true for *A. baumannii* relative proportion. However, the significant decrease in the proportion of MRSA isolates mentioned above is visible in this figure, along with the rise in the proportions of *E. cloacae* complex and *C. freundii* isolates starting in 2020.

Isolates of *C. freundii*, *E. cloacae* complex, *K. pneumoniae*, other enterobacterales, and *A. baumannii* were significantly more frequent beyond 48 h post-admission while *E. coli* and *S. aureus* isolates were mainly recovered within 48 h post-admission (Table 2). When carbapenemase-producing (CP) isolates are concerned, CP *E. coli* isolates displayed a significantly higher proportion within the first 48 h post-admission while CP *E. cloacae* complex isolates were more frequent beyond 48 h post-admission.

**Figure 5:**
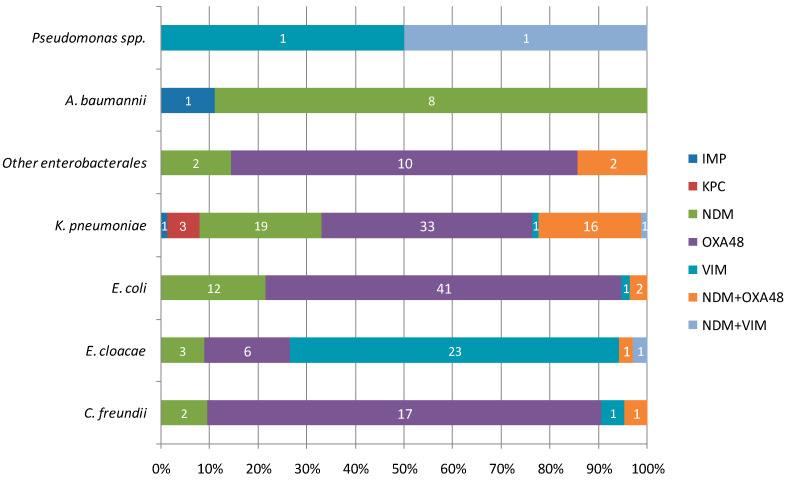
Distribution of carbapenemases in the various Gram-negative species. Numbers in white represent the number of isolates carrying the mentioned carbapenemase.

The number of isolates carrying the various carbapenemases per Gram-negative species is reported in Figure 5.

### 2.4. MDR Bacteria Isolation According to the Type of Hospitalization Ward

Overall, 1597 (56.2%) MDR bacteria were found in patients admitted to high-risk wards and 1245 (43.8%) to medium/low-risk ones. Time to first positive screening sample was significantly lower in medium/low-risk wards as compared to high-risk ones, with median values of 3 (interquartile range [1–9]) and 4 days (interquartile range [2–13]), respectively (Mann–Whitney test, *p* = 0.0001).The median carriage length was of 14 days (interquartile range [7–31]) in high-risk wards as compared to 22 days in medium/low-risk ones (Mann–Whitney test, *p* < 0.0001).

Proportions of MDR bacteria isolated from patients admitted to high-risk wards were significantly higher for *E. cloacae* complex and *S. aureus*. On the contrary, the proportions of *C. freundii* and *E. coli* were higher in medium/low-risk wards. No significant differences between wards were found for *A. baumannii* and *K. pneumoniae* proportions.

Times to the first positive screening sample for the 6 most prevalent species of MDR bacteria are detailed in Table 3 according to the ward type. Interestingly, *E. coli* and *S. aureus* shared the lowest median time to first positive screening, whatever the ward type. No significant difference in time to the first positive screening sample was found whatever the type of ward for both those species as well as for *C. freundii* and *E. cloacae* complex. However, a trend toward a higher time to first positive screening for *E. cloacae* complex was observed in high-risk wards (*p* = 0.0949, Mann–Whitney test). The overall *A. baumannii* time to first positive screening was the highest of all species studied. Moreover, it was significantly higher in high-risk wards as compared to medium/low-risk ones (Table 3). A higher time to first positive screening was also found in high-risk wards for *K. pneumoniae* (Table 3).

**Figure 6:**
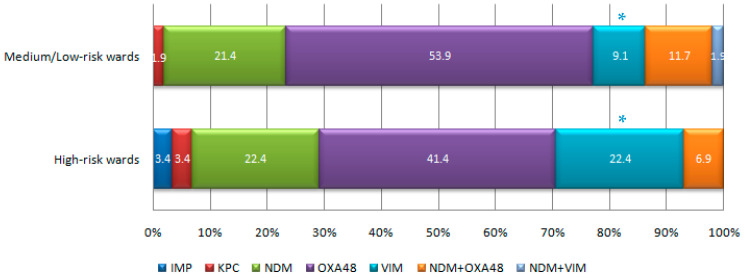
Relative proportions of the various carbapenemases encountered in high- and medium/low-risk wards (The total numbers of carbapenemases detected in high- and medium/low-risk wards were 58 and 154, respectively). Significant difference between high- and medium/low-risk wards at * *p* = 0.0095 (Z-ratio).

As for carbapenemase distribution between high- and medium/low-risk wards, the proportion of VIM carbapenemase was found to be significantly higher in high-risk ones (Figure 6).

## 3. Discussion

The first fact that could be highlighted from the results of this study was that no significant impact of the COVID-19 pandemic on the overall number of MDR-carrying patients or on the number of MDR isolates could be demonstrated, even though a slight reduction was noticeable for both in 2020 and 2021. This small reduction could be due to a stricter application of IPC measures by healthcare staff during the pandemic, hence limiting the transmission of MDR bacteria between patients over this period. It was no longer the case in 2022, hinting toward a return to a pre-COVID-19 level of application of IPC measures. Similarly, no change in time to the first positive screening sample or carriage duration could be highlighted in the statistical analysis. The only significant variations that could be put forward in relation to the COVID-19 pandemic were (i) the rise in both *C. freundii* and *E. cloacae* complex relative proportions among the MDR isolates starting in 2020 and (ii) the emergence of *K. pneumoniae* strains simultaneously producing two carbapenemases (OXA48 and NDM) post-pandemic. A few isolates were recovered in 2020 and 2021 but the spread of this carbapenemase combination exploded in 2022, possibly liked with a clonal dissemination of the original strain. In the future, it would be interesting to sequence representative isolates of such strains to better describe them and get a deeper understanding of their dissemination pattern. A similar rise in isolates producing multiple carbapenemases has been reported in three hospitals in Croatia post-COVID-19, with the same OXA48/NDM being the most frequent [15]. This might be preoccupying as these strains are extremely drug-resistant and could be the cause of therapeutic dead-ends in the near future.

When species are considered, the main contributors in positive screening samples were *E. coli* and *K. pneumoniae*, which is in accordance with most reports on MDR bacteria screenings in Europe and worldwide [16,17,18]. Their relative proportions among MDR bacteria detected by screening were stable over time. The rise in the proportion of *E. cloacae* complex recovered from screenings also fits the trend of an emerging nosocomial pathogen witnessed in other reports for this group of species [18,19]. Carbapenemase-producing *C. freundii* could be in the process of joining the emerging nosocomial pathogen club as their proportion also significantly increased in our study starting in 2020. Agreeing reports coming from various geographical regions back this claim up [19,20,21]. Carbapenemase-producing *P. aeruginosa* strains were seldom isolated over the 5-year screening period. Starting in 2018, a decrease in carbapenem-resistant *A. baumannii* (CRAB) isolation from screening samples occurred after several years with high incidence levels in both positive screenings and infections with CRAB in our hospital [22]. No more significant variations in CRAB numbers were registered over the 2018–2022 period but a new variant producing NDM carbapenemase instead of the sole OXA23 emerged. As for Gram-positive MDR bacteria, MRSA relative proportion amongst MDR bacteria significantly dropped from 2018 to 2022, which is in line with the reduction in MRSA witnessed in infections in the European region [16]. Finally, Vancomycin-Resistant Enterococci (VRE) were rarely encountered in this study.

The prevalence of MDR bacteria carriage at hospital admission has been depicted as considerable in a recent review [8]. Most studies included in this review were based on targeted approaches for screening such as those employed in our setting (screening if admission to high-risk wards or identification as high-risk patient). In our case, the short time to the first positive screening sample for both MDR *E. coli* and MRSA (whatever the type of ward) points toward community acquisition for these bacteria and/or for the acquisition of these microorganisms by patients during a previous hospital stay. Indeed, the global prevalence of ESBL-producing *E. coli* was recently computed to be 17.6% in the community settings, with figures dropping to 6% for the European region [23]. Moreover, a recent meta-analysis showed that the median ESBLE carriage by discharged patients was of 6 months and could reach 12 months in approximately a quarter of the cases. These patients were also a cause of dissemination of ESBLE in the community, as up to one-third of contacts from discharged patients could acquire a similar ESBLE [24]. Other species displayed times to first positive screening consistent with a hospital-acquired carriage, coining MDR *C. freundii*, *E. cloacae* complex, *K. pneumoniae*, other enterobacterales, and *A. baumannii* as nosocomial pathogens.

Regarding the part played by CP-microorganisms in MDR bacteria, a significant increase in the number of CPE amongst MDR isolates was witnessed over time, which is preoccupying but in agreement with various other recent reports [16,18,25,26]. Moreover, as reported by others, *K. pneumoniae* was the main contributor to CPE followed by *E. coli* [27]. Strains belonging to *E. cloacae* complex are now regularly emerging as the third category of CPE with their proportion steadily rising [25,26], which we also witnessed in our study with the following difference: the main carbapenemase produced by our strains was VIM and not the OXA48 or OXA48-like ones, contrary to what was previously described in Ireland and England [19,25]. However, this difference might be due to a documented outbreak in *E. cloacae* complex VIM isolates which started in 2020 in one of the ICU wards of the investigated hospital [28]. Nevertheless, VIM-carrying *E. cloacae* complex strains were also predominant in a study from Germany [29] and an isolate from Spain has also recently been reported [26], meaning that geographical differences in clone dissemination might also be at the root of this difference. Indeed, geographical differences are also seen in the distribution of other carbapenemases in CPE isolates, such as OXA48. This carbapenemase was the most frequently identified in our study, which is in accordance with larger datasets in Europe [16,19,25,26] but not in other regions such as Africa, Asia, and America where NDM and KPC are more prevalent [17,18]. Nevertheless, as discussed previously, the main concern regarding the type of carbapenemases produced by our CPE isolates remained the rise in the production of multiple enzymes by a single strain.

To conclude, it must be pointed out that his study has acknowledged limits, the first of which is that it is a single-center study. Therefore, even though most trends fit with larger reported datasets, results obtained here might only be representative of the local epidemiology of MDR bacteria. Secondly, as data were gathered retrospectively from banked laboratory results, no further genotyping of ESBLE or CPE was performed. Hence, values computed for carriage length were based on the recurring isolation of a bacterium with the same species/type of resistance combination for a given patient over time, which might not be accurate. The routine phenotypic screening for carbapenemase production focused on the five most frequent carbapenemases, i.e., OXA48, NDM, VIM, KPC, and IMP [25]. CPE strains generating other carbapenemases and/or variants of the five main carbapenemases not detected by the genotypic and/or immunochromatographic tests used here might therefore have eluded our census. Other shortcomings include the lack of information on previous hospitalization and/or recent antibiotic treatments for the patients included in the study. These items were not included in the research protocol and neither was the previous and/or subsequent occurrence of MDR infections in patients with a positive screening for MDR bacteria. It would therefore be interesting to expand the work to include these parameters and evaluate their impact.

## 4. Materials and Methods

### 4.1. Study Design and Data Collection

The study design was monocentric and retrospective on data gathered through the usual standard of care for patients hospitalized in the Centre Hospitalier Universitaire (CHU) Amiens-Picardie. The CHU Amiens-Picardie is a tertiary hospital comprising over 1600 beds dispatched between general medicine (including obstetrics) wards, intensive care units/wards, and long-term stay units for the elderly. Data between 1 January 2018 and 31 December 2022 were extracted from the computerized records of the Department of Hygiene, Biologic Risk and Environment as well as of the Department of Infectious Risk Prevention. The variables investigated included demographic information (birth date) as well as laboratory/clinical characteristics (ward, admission date, type of MDR screening, screening date, first positive screening date, isolated species, antibiotic resistance genes detected, and MDR status). All patients with a positive MDR screening were eligible for inclusion. The sole exclusion criterion was the lack of patient consent for the anonymous exploitation of their data. To avoid skewing in the calculation of yearly and global prevalences, only the first occurrence of a given MDR bacterium was retained for each patient for a given year.

This study was approved by the local ethics commission and registered under the number PI2023_843_0100.

### 4.2. MDR Screening Policy

MDR screenings were carried out following the French national recommendations for healthcare settings [13]. Nasal and cutaneous swabs were used for the detection of MRSA and seeded on Chromid^®^ MRSA agar (Biomérieux, Marcy l’Etoile, France). Rectal swabs were used and seeded on Chromid^®^ Carba Smart agar (Biomérieux), Drigalski with ceftazidime agar (Becton Dickinson, Le-Pont-de-Claix, France), and Chromid^®^ VRE (Biomérieux) to detect CPE, ESBLE, and VRE, respectively. Identification of the MDR isolates was performed using matrix-assisted laser desorption ionization time-of-flight mass spectrometry (MALDI Biotyper 2.2; Bruker Daltonik GmbH, Bremen, Germany). Carbapenemase production was ascertained using the Xpert^®^carba-R kit (Cepheid, Sunnyvale, CA, USA) and/or the Resist-5 OOKNV^®^ immunoassay kit (Coris Bioconcept, Gembloux, Belgium)and/or the NG-test CARBA-5^®^ immunoassay kit (Eurobioscientific, Les Ullis, France) and/or the OXA-23 K-SeT^®^ immunoassay kit (Coris Bioconcept, Gembloux, Belgium). Typically, immunoassay kits were the first line of tests used for carbapenemase detection. The Xpert^®^ carba-Rkit was implemented for patients identified as returning from travel abroad or in case of doubtful results obtained with immunoassay kits. VanA/vanB genes were detected using the Xpert^®^ vanA/vanB kit (Cepheid).

### 4.3. Definitions and Outcomes

To effectively implement the French recommendations to limit the spread of MDR in healthcare facilities [12], in the screening context of our hospital, an isolate was classified as MDR when it was identified as being (i) methicillin-resistant for *S. aureus* strains, (ii) vancomycin-resistant for *E. faecalis* strains, or (iii) an extended-spectrum beta-lactamase and/or a carbapenemase producer for enterobacteria and other Gram-negative bacteria. No further MIC determination was carried out in the routinely held MDR screening.

For a given bacterium/patient pair, time to positive screening was defined as the time elapsed between the admission date of the patient and the date of the first screening sample found positive for this bacterium by the Department of Hygiene, Biologic Risk and Environment. Length of carriage was defined as the time elapsed between the first and the last positive screening samples on record for a given bacterium/patient pair.

To discriminate patients walking into the hospital already carrying MDR bacteria and patients with hospital-acquired MDR carriage, similarly to what is commonly accepted for hospital-acquired infections (HAIs), a cut-off of 2 days post-admission was set to discriminate nosocomial carriage (i.e., patients with positive screening samples registered from 3 days post-admission and onwards were classified as having acquired the bacterium in the hospital) from community-acquired carriage.

The classification between high-risk and low/medium-risk wards was based on a previously described methodology [8]. More specifically, wards classified as high-risk were ICUs or multiple wards including an ICU as well as hematology, transplant, rehabilitation, and burn units. All other wards were classified as low/medium risk.

### 4.4. Statistical Analysis

The Kolgromov–Smirnov test was performed to assess whether the observed proportions of (i) MDR strains and (ii) positive patients retrieved by year differed from a uniform distribution over the 5 years of the study. It was also used to assess whether the observed proportions of ESBLE, CPE, other MDR Gram-negative bacteria, VRE, and MRSA retrieved by year were significantly different from the global proportions calculated over the 5-year period for each type of MDR bacteria. The z-ratio for the difference between two independent proportions, Pearson’s Chi-square test or Fisher’s exact test was employed to compare independent proportions, depending on the sample size. Mann–Whitney test was employed to compare time to positivity and length of carriage values between two conditions (e.g., between high-risk and medium/low-risk wards or two bacterial species). Kruskal–Wallis test was performed to compare time to positivity and length of carriage values over more than two conditions (e.g., the five years of the study or more than two bacterial species). Statistical significance was inferred for a *p*-value below 0.05.

## Figures and Tables

**Table 1:** Overall number of positive patients, time to first positive screening sample, and length of carriage.

Year	Patients Number (%)	Time to First Positive Screening (Days)	Length of Carriage (Days)
2018	534 (21.9)	3 [1–10] ^1^	15 [7–34.75] ^1^
2019	570 (23.3)	3 [1–11]	14 [7–35]
2020	457 (18.7)	4 [1–13]	14.5 [7–35.75]
2021	420 (17.2)	3 [1–10]	13 [7–35]
2022	461(18.9)	4 [2–12]	15 [7–45.75]
Total	2442 (100)	3 [1–11]	19 [7–70]

^1^ Expressed as median (interquartile range).

**Table 2:** Proportions of MDR and carbapenemase-producing MDR isolates per species according to the time to first positive screening sample.

Species	Time to Positivity ^1^		Total ^1^
	≤48 h	>48 h	
*C. freundii*	1.6 * (7.2)	3.3 * (11.4)	2.6 (10.3)
*E. cloacae* complex	4.0 ** (7.2 ^$^)	11.4 ** (18.8 ^$^)	8.4 (15.7)
*E. coli*	54.1 ** (40 ^£^)	30.9 ** (20.1 ^£^)	40.4 (25.5)
*K. pneumoniae*	21.4 ** (36.4)	37.1 ** (34.9)	30.6 (35.3)
Other enterobacterales	2.2 *** (3.6)	4.1 *** (9.4)	3.3 (7.8)
Total enterobacterales	83.3 ^†^ (94.5)	86.7 ^†^ (94.6)	85.3 (94.6)
*A. baumannii* ^2^	1.0 ** (1.8)	4.0 ** (5.4)	2.8 (4.4)
*P. aeruginosa*	0.1 (1.8)	0.1 (0)	0.1 (0.5)
*P. putida*	0.1 (1.8)	0 (0)	0.1 (0.5)
*E. faecium*	0.2	0.2	0.2
*S. aureus*	15.3 **	8.9 **	11.5
Total	100 (100)	100 (100)	100 (100)

^1^ Overall proportion (proportion of carbapenemase-producing isolates) expressed as percentages; ^2^ As nearly all MDR *A. baumannii* isolates from 2018 and beyond harbored OXA-23 carbapenemase, only the carriage of other carbapenemases is reported in the table for this species. Significant difference between proportions up to 48 h and after 48 h (Z-ratio) at: * *p* = 0.0043; ** *p* < 0.0002; *** *p* = 0065; ^†^ *p* = 0.0107; ^£^ *p* = 0.0039. Significant differences between proportions (Pearson’s Chi-square) at ^$^ *p* = 0.0447.

**Table 3:** Time to first positive screening sample according to the ward type and species.

	Species	Proportion (*n*) ^a^	Median ^b^	Interquartile Range ^b^
All wards	*Acinetobacter baumannii*	2.8 (80)	14.5	6–25.25
	*Citrobacter freundii*	2.6 (73)	10	3–15
	*Enterobacter cloacae* complex	8.4 (238)	10	3–23
	*Escherichia coli*	40.4 (1148)	2	1–5
	*Klebsiella pneumoniae*	30.8 (875)	7	2–16
	*Staphylococcus aureus*	11.5 (327)	2	1–4
High-risk wards	*Acinetobacter baumannii*	3.3 (53)	18 *	8–29
	*Citrobacter freundii*	1.6 (26) ^†^	10.5	2–19.25
	*Enterobacter cloacae* complex	10.0 (159) ^‡^	11	4–23
	*Escherichia coli*	36.4 (581) ^£^	2	1–6
	*Klebsiella pneumoniae*	31.6 (505)	8 **	3–17
	*Staphylococcus aureus*	13.5 (216) ^£^	2	1–4
Medium/Low-risk wards	*Acinetobacter baumannii*	2.2 (27)	8 *	3.5–17
	*Citrobacter freundii*	3.8 (47) ^†^	8	3–14.5
	*Enterobacter cloacae* complex	6.3 (79) ^‡^	8	2–18
	*Escherichia coli*	45.5 (567) ^£^	2	1–5
	*Klebsiella pneumoniae*	29.7 (370)	5 **	2–13.75
	*Staphylococcus aureus*	8.9 (111) ^£^	2	1–4.5

^a^: expressed as percentage of the overall number of MDR isolates for the considered type of ward; *n* = number of isolates; ^b^: expressed in days. Significant difference between proportions of the isolated species in high-risk and medium/low-risk wards (z-ratio) at ^†^: *p* = 0.0003; ^‡^: *p* = 0.0006; ^£^: *p* < 0.0002. Significant difference between median time to first positive screening sample in high-risk and medium/low-risk wards (Mann–Whitney test) at *: *p* = 0.0117; **: *p* = 0.0007.

## Data Availability

The datasets created and analyzed during the current study are not publicly available because they contain patient information that was collected under the responsibility of D.L. for internal use only. These data are not to be disclosed publicly as per the French legislation on clinical trials and the European GDPR.

## Supplementary Materials

The following supporting information can be downloaded at: https://www.mdpi.com/article/10.3390/antibiotics12081314/s1, Table S1: List of first isolates recovered over the 2018–2022 period.
