# Supplementary material for: Trends in Antibiotic-Resistant Bacteria Isolated from Screening Clinical Samples in a Tertiary Care Hospital over the 2018–2022 Period
Source: Antibiotics (Basel). 2023 Aug 14;12(8):1314. doi: 10.3390/antibiotics12081314 (PMC10451239; doi:10.3390/antibiotics12081314)
Supplement: Supplementary file 1 [file antibiotics-12-01314-s001.zip › antibiotics-2535609-supplementary.pdf]

Isolate Number	Species	Resistance
1	*Enterobacter cloacae complex*	ESBL
2	*Escherichia coli*	ESBL
3	*Klebsiella pneumoniae*	ESBL
4	*Enterobacter cloacae complex*	ESBL
5	*Escherichia coli*	OXA48
6	*Escherichia coli*	ESBL
7	*Escherichia coli*	ESBL
8	*Enterobacter cloacae complex*	ESBL
9	*Klebsiella pneumoniae*	ESBL
10	*Enterobacter cloacae complex*	ESBL
11	*Klebsiella pneumoniae*	ESBL
12	*Klebsiella pneumoniae*	ESBL
13	*Staphylococcus aureus*	MRSA
14	*Escherichia coli*	ESBL
15	*Klebsiella oxytoca*	NDM
16	*Klebsiella aerogenes*	NDM
17	*Escherichia coli*	ESBL
18	*Enterobacter cloacae complex*	ESBL
19	*Klebsiella pneumoniae*	ESBL
20	*Escherichia coli*	ESBL
21	*Klebsiella pneumoniae*	ESBL
22	*Klebsiella pneumoniae*	ESBL
23	*Staphylococcus aureus*	MRSA
24	*Escherichia coli*	ESBL
25	*Klebsiella pneumoniae*	ESBL
26	*Klebsiella pneumoniae*	ESBL
27	*Klebsiella aerogenes*	ESBL
28	*Escherichia coli*	ESBL
29	*Staphylococcus aureus*	MRSA
30	*Escherichia coli*	ESBL
31	*Citrobacter freundii*	ESBL
32	*Klebsiella oxytoca*	ESBL
33	*Staphylococcus aureus*	MRSA
34	*Escherichia coli*	ESBL
35	*Escherichia coli*	ESBL
36	*Escherichia coli*	ESBL
37	*Klebsiella pneumoniae*	ESBL
38	*Klebsiella pneumoniae*	ESBL
39	*Klebsiella pneumoniae*	ESBL
40	*Escherichia coli*	ESBL
41	*Enterobacter cloacae complex*	ESBL
42	*Citrobacter freundii*	ESBL
43	*Escherichia coli*	ESBL
44	*Escherichia coli*	ESBL
45	*Escherichia coli*	ESBL

46	*Escherichia coli*	ESBL
47	*Escherichia coli*	ESBL
48	*Escherichia coli*	ESBL
49	*Klebsiella pneumoniae*	ESBL
50	*Escherichia coli*	ESBL
51	*Staphylococcus aureus*	MRSA
52	*Escherichia coli*	ESBL
53	*Escherichia coli*	ESBL
54	*Klebsiella pneumoniae*	ESBL
55	*Staphylococcus aureus*	MRSA
56	*Enterobacter cloacae complex*	ESBL
57	*Escherichia coli*	ESBL
58	*Citrobacter freundii*	ESBL
59	*Escherichia coli*	ESBL
60	*Escherichia coli*	ESBL
61	*Escherichia coli*	ESBL
62	*Acinetobacter baumannii*	OXA23
63	*Enterobacter cloacae complex*	ESBL
64	*Klebsiella pneumoniae*	ESBL
65	*Klebsiella pneumoniae*	ESBL
66	*Klebsiella pneumoniae*	ESBL
67	*Klebsiella pneumoniae*	ESBL
68	*Klebsiella oxytoca*	ESBL
69	*Klebsiella pneumoniae*	ESBL
70	*Staphylococcus aureus*	MRSA
71	*Escherichia coli*	ESBL
72	*Escherichia coli*	ESBL
73	*Klebsiella pneumoniae*	ESBL
74	*Staphylococcus aureus*	MRSA
75	*Klebsiella pneumoniae*	ESBL
76	*Klebsiella aerogenes*	ESBL
77	*Escherichia coli*	ESBL
78	*Escherichia coli*	ESBL
79	*Klebsiella pneumoniae*	ESBL
80	*Escherichia coli*	ESBL
81	*Staphylococcus aureus*	MRSA
82	*Staphylococcus aureus*	MRSA
83	*Klebsiella pneumoniae*	ESBL
84	*Citrobacter koseri*	ESBL
85	*Escherichia coli*	ESBL
86	*Escherichia coli*	ESBL
87	*Escherichia coli*	ESBL
88	*Klebsiella pneumoniae*	ESBL
89	*Escherichia coli*	ESBL
90	*Escherichia coli*	ESBL
91	*Enterobacter cloacae complex*	ESBL

92	*Escherichia coli*	ESBL
93	*Escherichia coli*	ESBL
94	*Escherichia coli*	ESBL
95	*Staphylococcus aureus*	MRSA
96	*Staphylococcus aureus*	MRSA
97	*Staphylococcus aureus*	MRSA
98	*Klebsiella pneumoniae*	ESBL
99	*Enterobacter cloacae complex*	ESBL
100	*Raoultella ornithinolytica*	ESBL
101	*Enterobacter cloacae complex*	ESBL
102	*Escherichia coli*	ESBL
103	*Escherichia coli*	ESBL
104	*Klebsiella pneumoniae*	ESBL
105	*Morganella morganii*	ESBL
106	*Staphylococcus aureus*	MRSA
107	*Staphylococcus aureus*	MRSA
108	*Enterobacter cloacae complex*	ESBL
109	*Escherichia coli*	ESBL
110	*Escherichia coli*	ESBL
111	*Escherichia coli*	ESBL
112	*Klebsiella pneumoniae*	ESBL
113	*Escherichia coli*	ESBL
114	*Klebsiella pneumoniae*	ESBL
115	*Escherichia coli*	ESBL
116	*Escherichia coli*	ESBL
117	*Escherichia coli*	ESBL
118	*Escherichia coli*	ESBL
119	*Klebsiella pneumoniae*	ESBL
120	*Morganella morganii*	ESBL
121	*Staphylococcus aureus*	MRSA
122	*Enterobacter cloacae complex*	ESBL
123	*Klebsiella pneumoniae*	ESBL
124	*Klebsiella pneumoniae*	ESBL
125	*Enterobacter cloacae complex*	ESBL
126	*Staphylococcus aureus*	MRSA
127	*Klebsiella pneumoniae*	ESBL
128	*Klebsiella pneumoniae*	ESBL
129	*Escherichia coli*	ESBL
130	*Escherichia coli*	ESBL
131	*Citrobacter freundii*	ESBL
132	*Klebsiella pneumoniae*	ESBL
133	*Klebsiella pneumoniae*	ESBL
134	*Klebsiella pneumoniae*	ESBL
135	*Escherichia coli*	ESBL
136	*Klebsiella pneumoniae*	ESBL
137	*Escherichia coli*	OXA48

138	*Klebsiella pneumoniae*	OXA48
139	*Serratia marcescens*	OXA48
140	*Klebsiella pneumoniae*	ESBL
141	*Escherichia coli*	ESBL
142	*Klebsiella pneumoniae*	ESBL
143	*Escherichia coli*	ESBL
144	*Klebsiella pneumoniae*	ESBL
145	*Escherichia coli*	ESBL
146	*Escherichia coli*	ESBL
147	*Escherichia coli*	ESBL
148	*Klebsiella pneumoniae*	ESBL
149	*Escherichia coli*	ESBL
150	*Staphylococcus aureus*	MRSA
151	*Klebsiella pneumoniae*	ESBL
152	*Escherichia coli*	ESBL
153	*Acinetobacter baumannii*	OXA23
154	*Klebsiella pneumoniae*	ESBL
155	*Escherichia coli*	ESBL
156	*Staphylococcus aureus*	MRSA
157	*Enterobacter cloacae complex*	VIM
158	*Escherichia coli*	ESBL
159	*Klebsiella pneumoniae*	ESBL
160	*Enterobacter cloacae complex*	ESBL
161	*Klebsiella pneumoniae*	ESBL
162	*Klebsiella pneumoniae*	ESBL
163	*Acinetobacter baumannii*	NDM
164	*Citrobacter braakii*	ESBL
165	*Staphylococcus aureus*	MRSA
166	*Enterobacter cloacae complex*	ESBL
167	*Enterobacter cloacae complex*	ESBL
168	*Escherichia coli*	ESBL
169	*Escherichia coli*	ESBL
170	*Klebsiella pneumoniae*	ESBL
171	*Klebsiella oxytoca*	ESBL
172	*Klebsiella pneumoniae*	ESBL
173	*Enterobacter cloacae complex*	VIM
174	*Enterobacter cloacae complex*	ESBL
175	*Escherichia coli*	ESBL
176	*Escherichia coli*	ESBL
177	*Staphylococcus aureus*	MRSA
178	*Staphylococcus aureus*	MRSA
179	*Staphylococcus aureus*	MRSA
180	*Klebsiella pneumoniae*	ESBL
181	*Klebsiella pneumoniae*	ESBL
182	*Klebsiella pneumoniae*	ESBL
183	*Escherichia coli*	ESBL

184	*Escherichia coli*	ESBL
185	*Staphylococcus aureus*	MRSA
186	*Staphylococcus aureus*	MRSA
187	*Enterobacter cloacae complex*	ESBL
188	*Klebsiella pneumoniae*	ESBL
189	*Escherichia coli*	ESBL
190	*Klebsiella pneumoniae*	ESBL
191	*Staphylococcus aureus*	MRSA
192	*Klebsiella pneumoniae*	ESBL
193	*Staphylococcus aureus*	MRSA
194	*Klebsiella pneumoniae*	ESBL
195	*Klebsiella pneumoniae*	ESBL
196	*Staphylococcus aureus*	MRSA
197	*Staphylococcus aureus*	MRSA
198	*Klebsiella pneumoniae*	ESBL
199	*Escherichia coli*	ESBL
200	*Escherichia coli*	ESBL
201	*Escherichia coli*	ESBL
202	*Staphylococcus aureus*	MRSA
203	*Escherichia coli*	ESBL
204	*Citrobacter freundii*	ESBL
205	*Enterobacter cloacae complex*	ESBL
206	*Escherichia coli*	ESBL
207	*Klebsiella pneumoniae*	ESBL
208	*Klebsiella pneumoniae*	ESBL
209	*Escherichia coli*	ESBL
210	*Klebsiella pneumoniae*	ESBL
211	*Staphylococcus aureus*	MRSA
212	*Klebsiella pneumoniae*	ESBL
213	*Klebsiella pneumoniae*	ESBL
214	*Escherichia coli*	ESBL
215	*Escherichia coli*	ESBL
216	*Klebsiella pneumoniae*	ESBL
217	*Escherichia coli*	ESBL
218	*Escherichia coli*	ESBL
219	*Klebsiella pneumoniae*	ESBL
220	*Escherichia coli*	ESBL
221	*Escherichia coli*	ESBL
222	*Acinetobacter baumannii*	NDM
223	*Escherichia coli*	ESBL
224	*Klebsiella pneumoniae*	ESBL
225	*Escherichia coli*	ESBL
226	*Enterobacter cloacae complex*	ESBL
227	*Leclercia adecarboxylata*	ESBL
228	*Escherichia coli*	ESBL
229	*Staphylococcus aureus*	MRSA

230	*Enterobacter cloacae complex*	ESBL
231	*Klebsiella pneumoniae*	ESBL
232	*Klebsiella pneumoniae*	ESBL
233	*Escherichia coli*	ESBL
234	*Escherichia coli*	ESBL
235	*Klebsiella pneumoniae*	ESBL
236	*Enterobacter cloacae complex*	ESBL
237	*Citrobacter braakii*	ESBL
238	*Escherichia coli*	ESBL
239	*Escherichia coli*	ESBL
240	*Klebsiella pneumoniae*	ESBL
241	*Acinetobacter baumannii*	OXA23
242	*Staphylococcus aureus*	MRSA
243	*Escherichia coli*	ESBL
244	*Escherichia coli*	ESBL
245	*Escherichia coli*	ESBL
246	*Acinetobacter baumannii*	OXA23
247	*Enterobacter cloacae complex*	ESBL
248	*Klebsiella pneumoniae*	ESBL
249	*Escherichia coli*	ESBL
250	*Escherichia coli*	ESBL
251	*Escherichia coli*	ESBL
252	*Staphylococcus aureus*	MRSA
253	*Escherichia coli*	ESBL
254	*Escherichia coli*	OXA48
255	*Escherichia coli*	ESBL
256	*Klebsiella pneumoniae*	ESBL
257	*Acinetobacter baumannii*	OXA23
258	*Klebsiella pneumoniae*	ESBL
259	*Klebsiella pneumoniae*	ESBL
260	*Escherichia coli*	ESBL
261	*Escherichia coli*	ESBL
262	*Escherichia coli*	ESBL
263	*Escherichia coli*	ESBL
264	*Acinetobacter baumannii*	OXA23
265	*Klebsiella pneumoniae*	ESBL
266	*Escherichia coli*	ESBL
267	*Escherichia coli*	ESBL
268	*Escherichia coli*	ESBL
269	*Klebsiella pneumoniae*	ESBL
270	*Staphylococcus aureus*	MRSA
271	*Klebsiella pneumoniae*	ESBL
272	*Escherichia coli*	ESBL
273	*Staphylococcus aureus*	MRSA
274	*Enterobacter cloacae complex*	ESBL
275	*Citrobacter freundii*	ESBL

276	*Staphylococcus aureus*	MRSA
277	*Staphylococcus aureus*	MRSA
278	*Staphylococcus aureus*	MRSA
279	*Escherichia coli*	ESBL
280	*Escherichia coli*	ESBL
281	*Klebsiella pneumoniae*	KPC
282	*Staphylococcus aureus*	MRSA
283	*Escherichia coli*	ESBL
284	*Acinetobacter baumannii*	OXA23
285	*Klebsiella pneumoniae*	ESBL
286	*Klebsiella pneumoniae*	ESBL
287	*Escherichia coli*	ESBL
288	*Klebsiella pneumoniae*	ESBL
289	*Escherichia coli*	ESBL
290	*Staphylococcus aureus*	MRSA
291	*Klebsiella pneumoniae*	ESBL
292	*Escherichia coli*	ESBL
293	*Acinetobacter baumannii*	OXA23
294	*Escherichia coli*	ESBL
295	*Klebsiella pneumoniae*	ESBL
296	*Klebsiella pneumoniae*	ESBL
297	*Enterobacter cloacae complex*	ESBL
298	*Klebsiella pneumoniae*	ESBL
299	*Escherichia coli*	ESBL
300	*Klebsiella pneumoniae*	ESBL
301	*Escherichia coli*	ESBL
302	*Klebsiella pneumoniae*	ESBL
303	*Klebsiella pneumoniae*	ESBL
304	*Staphylococcus aureus*	MRSA
305	*Escherichia coli*	ESBL
306	*Klebsiella pneumoniae*	ESBL
307	*Klebsiella oxytoca*	ESBL
308	*Klebsiella pneumoniae*	ESBL
309	*Klebsiella pneumoniae*	ESBL
310	*Staphylococcus aureus*	MRSA
311	*Escherichia coli*	ESBL
312	*Klebsiella pneumoniae*	ESBL
313	*Escherichia coli*	ESBL
314	*Escherichia coli*	ESBL
315	*Escherichia coli*	ESBL
316	*Escherichia coli*	ESBL
317	*Klebsiella pneumoniae*	ESBL
318	*Enterobacter cloacae complex*	ESBL
319	*Klebsiella pneumoniae*	ESBL
320	*Staphylococcus aureus*	MRSA
321	*Enterobacter cloacae complex*	ESBL

322	*Escherichia coli*	ESBL
323	*Escherichia coli*	ESBL
324	*Klebsiella pneumoniae*	ESBL
325	*Escherichia coli*	ESBL
326	*Klebsiella pneumoniae*	ESBL
327	*Klebsiella pneumoniae*	ESBL
328	*Klebsiella pneumoniae*	KPC
329	*Escherichia coli*	ESBL
330	*Staphylococcus aureus*	MRSA
331	*Klebsiella pneumoniae*	ESBL
332	*Klebsiella pneumoniae*	ESBL
333	*Klebsiella pneumoniae*	ESBL
334	*Klebsiella pneumoniae*	ESBL
335	*Klebsiella pneumoniae*	ESBL
336	*Staphylococcus aureus*	MRSA
337	*Escherichia coli*	ESBL
338	*Klebsiella pneumoniae*	ESBL
339	*Citrobacter freundii*	OXA48
340	*Klebsiella pneumoniae*	OXA48
341	*Staphylococcus aureus*	MRSA
342	*Klebsiella pneumoniae*	ESBL
343	*Klebsiella pneumoniae*	ESBL
344	*Escherichia coli*	ESBL
345	*Escherichia coli*	ESBL
346	*Klebsiella pneumoniae*	ESBL
347	*Escherichia coli*	ESBL
348	*Staphylococcus aureus*	MRSA
349	*Escherichia coli*	ESBL
350	*Klebsiella pneumoniae*	ESBL
351	*Staphylococcus aureus*	MRSA
352	*Enterobacter cloacae complex*	ESBL
353	*Escherichia coli*	ESBL
354	*Escherichia coli*	ESBL
355	*Klebsiella pneumoniae*	ESBL
356	*Escherichia coli*	ESBL
357	*Escherichia coli*	ESBL
358	*Klebsiella pneumoniae*	ESBL
359	*Escherichia coli*	ESBL
360	*Escherichia coli*	ESBL
361	*Escherichia coli*	ESBL
362	*Acinetobacter baumannii*	OXA23
363	*Enterobacter cloacae complex*	ESBL
364	*Staphylococcus aureus*	MRSA
365	*Escherichia coli*	ESBL
366	*Escherichia coli*	ESBL
367	*Escherichia coli*	ESBL

368	*Escherichia coli*	ESBL
369	*Citrobacter freundii*	ESBL
370	*Escherichia coli*	ESBL
371	*Klebsiella pneumoniae*	ESBL
372	*Klebsiella pneumoniae*	ESBL
373	*Escherichia coli*	ESBL
374	*Klebsiella pneumoniae*	ESBL
375	*Escherichia coli*	ESBL
376	*Escherichia coli*	ESBL
377	*Citrobacter freundii*	OXA48
378	*Morganella morganii*	ESBL
379	*Enterobacter cloacae complex*	ESBL
380	*Escherichia coli*	ESBL
381	*Escherichia coli*	ESBL
382	*Klebsiella pneumoniae*	ESBL
383	*Klebsiella pneumoniae*	ESBL
384	*Acinetobacter baumannii*	OXA23
385	*Enterobacter cloacae complex*	ESBL
386	*Escherichia coli*	ESBL
387	*Escherichia coli*	ESBL
388	*Staphylococcus aureus*	MRSA
389	*Staphylococcus aureus*	MRSA
390	*Klebsiella pneumoniae*	ESBL
391	*Klebsiella pneumoniae*	ESBL
392	*Klebsiella pneumoniae*	ESBL
393	*Escherichia coli*	ESBL
394	*Klebsiella pneumoniae*	ESBL
395	*Escherichia coli*	ESBL
396	*Klebsiella pneumoniae*	ESBL
397	*Escherichia coli*	ESBL
398	*Klebsiella pneumoniae*	ESBL
399	*Acinetobacter baumannii*	OXA23
400	*Klebsiella pneumoniae*	ESBL
401	*Klebsiella pneumoniae*	ESBL
402	*Enterobacter cloacae complex*	ESBL
403	*Morganella morganii*	ESBL
404	*Staphylococcus aureus*	MRSA
405	*Klebsiella pneumoniae*	ESBL
406	*Escherichia coli*	ESBL
407	*Staphylococcus aureus*	MRSA
408	*Klebsiella pneumoniae*	ESBL
409	*Klebsiella pneumoniae*	ESBL
410	*Klebsiella pneumoniae*	ESBL
411	*Enterobacter cloacae complex*	ESBL
412	*Escherichia coli*	ESBL
413	*Citrobacter koseri*	ESBL

414	*Escherichia coli*	ESBL
415	*Escherichia coli*	ESBL
416	*Klebsiella aerogenes*	ESBL
417	*Escherichia coli*	ESBL
418	*Klebsiella pneumoniae*	ESBL
419	*Citrobacter freundii*	OXA48
420	*Klebsiella pneumoniae*	ESBL
421	*Acinetobacter baumannii*	OXA23
422	*Enterobacter cloacae complex*	ESBL
423	*Acinetobacter baumannii*	OXA23
424	*Klebsiella pneumoniae*	ESBL
425	*Escherichia coli*	ESBL
426	*Staphylococcus aureus*	MRSA
427	*Escherichia coli*	ESBL
428	*Klebsiella pneumoniae*	ESBL
429	*Klebsiella pneumoniae*	ESBL
430	*Escherichia coli*	ESBL
431	*Enterobacter cloacae complex*	VIM
432	*Staphylococcus aureus*	MRSA
433	*Klebsiella pneumoniae*	ESBL
434	*Escherichia coli*	ESBL
435	*Klebsiella pneumoniae*	ESBL
436	*Enterobacter cloacae complex*	ESBL
437	*Staphylococcus aureus*	MRSA
438	*Staphylococcus aureus*	MRSA
439	*Escherichia coli*	ESBL
440	*Escherichia coli*	ESBL
441	*Enterobacter cloacae complex*	ESBL
442	*Klebsiella pneumoniae*	ESBL
443	*Enterobacter cloacae complex*	ESBL
444	*Staphylococcus aureus*	MRSA
445	*Escherichia coli*	ESBL
446	*Staphylococcus aureus*	MRSA
447	*Klebsiella pneumoniae*	ESBL
448	*Klebsiella pneumoniae*	ESBL
449	*Staphylococcus aureus*	MRSA
450	*Klebsiella pneumoniae*	ESBL
451	*Staphylococcus aureus*	MRSA
452	*Klebsiella pneumoniae*	ESBL
453	*Staphylococcus aureus*	MRSA
454	*Klebsiella pneumoniae*	ESBL
455	*Klebsiella pneumoniae*	ESBL
456	*Escherichia coli*	ESBL
457	*Acinetobacter baumannii*	OXA23
458	*Escherichia coli*	ESBL
459	*Escherichia coli*	ESBL

460	*Staphylococcus aureus*	MRSA
461	*Klebsiella pneumoniae*	ESBL
462	*Escherichia coli*	ESBL
463	*Escherichia coli*	ESBL
464	*Klebsiella pneumoniae*	ESBL
465	*Enterobacter cloacae complex*	ESBL
466	*Klebsiella oxytoca*	ESBL
467	*Escherichia coli*	ESBL
468	*Escherichia coli*	ESBL
469	*Klebsiella pneumoniae*	ESBL
470	*Klebsiella pneumoniae*	ESBL
471	*Escherichia coli*	ESBL
472	*Staphylococcus aureus*	MRSA
473	*Klebsiella pneumoniae*	OXA48
474	*Escherichia coli*	ESBL
475	*Escherichia coli*	ESBL
476	*Escherichia coli*	ESBL
477	*Klebsiella pneumoniae*	ESBL
478	*Klebsiella pneumoniae*	ESBL
479	*Acinetobacter baumannii*	OXA23
480	*Klebsiella pneumoniae*	ESBL
481	*Staphylococcus aureus*	MRSA
482	*Klebsiella pneumoniae*	ESBL
483	*Enterobacter cloacae complex*	ESBL
484	*Acinetobacter baumannii*	OXA23
485	*Klebsiella pneumoniae*	ESBL
486	*Escherichia coli*	ESBL
487	*Klebsiella pneumoniae*	ESBL
488	*Klebsiella pneumoniae*	ESBL
489	*Enterobacter cloacae complex*	ESBL
490	*Staphylococcus aureus*	MRSA
491	*Staphylococcus aureus*	MRSA
492	*Klebsiella pneumoniae*	ESBL
493	*Staphylococcus aureus*	MRSA
494	*Klebsiella pneumoniae*	ESBL
495	*Escherichia coli*	ESBL
496	*Escherichia coli*	ESBL
497	*Enterobacter cloacae complex*	ESBL
498	*Citrobacter freundii*	ESBL
499	*Escherichia coli*	ESBL
500	*Klebsiella pneumoniae*	ESBL
501	*Staphylococcus aureus*	MRSA
502	*Klebsiella pneumoniae*	ESBL
503	*Citrobacter freundii*	OXA48
504	*Enterobacter cloacae complex*	ESBL
505	*Escherichia coli*	ESBL

506	*Escherichia coli*	ESBL
507	*Acinetobacter baumannii*	OXA23
508	*Acinetobacter baumannii*	OXA23
509	*Escherichia coli*	ESBL
510	*Klebsiella pneumoniae*	ESBL
511	*Enterobacter cloacae complex*	ESBL
512	*Escherichia coli*	ESBL
513	*Staphylococcus aureus*	MRSA
514	*Escherichia coli*	ESBL
515	*Klebsiella pneumoniae*	ESBL
516	*Staphylococcus aureus*	MRSA
517	*Enterobacter cloacae complex*	ESBL
518	*Staphylococcus aureus*	MRSA
519	*Escherichia coli*	ESBL
520	*Escherichia coli*	ESBL
521	*Morganella morganii*	ESBL
522	*Escherichia coli*	ESBL
523	*Escherichia coli*	ESBL
524	*Klebsiella pneumoniae*	ESBL
525	*Klebsiella pneumoniae*	ESBL
526	*Staphylococcus aureus*	MRSA
527	*Escherichia coli*	ESBL
528	*Staphylococcus aureus*	MRSA
529	*Klebsiella pneumoniae*	ESBL
530	*Escherichia coli*	ESBL
531	*Morganella morganii*	ESBL
532	*Klebsiella pneumoniae*	ESBL
533	*Escherichia coli*	ESBL
534	*Escherichia coli*	ESBL
535	*Enterobacter cloacae complex*	ESBL
536	*Escherichia coli*	ESBL
537	*Escherichia coli*	ESBL
538	*Klebsiella pneumoniae*	ESBL
539	*Klebsiella pneumoniae*	ESBL
540	*Enterobacter cloacae complex*	ESBL
541	*Escherichia coli*	ESBL
542	*Staphylococcus aureus*	MRSA
543	*Klebsiella pneumoniae*	ESBL
544	*Escherichia coli*	ESBL
545	*Escherichia coli*	ESBL
546	*Staphylococcus aureus*	MRSA
547	*Klebsiella pneumoniae*	ESBL
548	*Escherichia coli*	ESBL
549	*Escherichia coli*	ESBL
550	*Enterobacter cloacae complex*	ESBL
551	*Escherichia coli*	ESBL

552	*Klebsiella pneumoniae*	ESBL
553	*Escherichia coli*	ESBL
554	*Enterobacter cloacae complex*	ESBL
555	*Escherichia coli*	ESBL
556	*Escherichia coli*	ESBL
557	*Escherichia coli*	ESBL
558	*Klebsiella pneumoniae*	ESBL
559	*Enterobacter cloacae complex*	VIM
560	*Escherichia coli*	ESBL
561	*Escherichia coli*	ESBL
562	*Escherichia coli*	ESBL
563	*Klebsiella pneumoniae*	ESBL
564	*Acinetobacter baumannii*	OXA23
565	*Escherichia coli*	ESBL
566	*Enterobacter cloacae complex*	ESBL
567	*Citrobacter freundii*	ESBL
568	*Staphylococcus aureus*	MRSA
569	*Klebsiella pneumoniae*	ESBL
570	*Escherichia coli*	ESBL
571	*Staphylococcus aureus*	MRSA
572	*Klebsiella pneumoniae*	ESBL
573	*Staphylococcus aureus*	MRSA
574	*Klebsiella pneumoniae*	ESBL
575	*Enterobacter cloacae complex*	ESBL
576	*Escherichia coli*	ESBL
577	*Klebsiella pneumoniae*	ESBL
578	*Escherichia coli*	ESBL
579	*Klebsiella pneumoniae*	ESBL
580	*Staphylococcus aureus*	MRSA
581	*Klebsiella pneumoniae*	ESBL
582	*Klebsiella pneumoniae*	ESBL
583	*Escherichia coli*	ESBL
584	*Escherichia coli*	ESBL
585	*Escherichia coli*	ESBL
586	*Escherichia coli*	ESBL
587	*Staphylococcus aureus*	MRSA
588	*Staphylococcus aureus*	MRSA
589	*Klebsiella pneumoniae*	ESBL
590	*Escherichia coli*	ESBL
591	*Escherichia coli*	ESBL
592	*Klebsiella pneumoniae*	ESBL
593	*Enterobacter cloacae complex*	OXA48
594	*Escherichia coli*	ESBL
595	*Escherichia coli*	ESBL
596	*Escherichia coli*	ESBL
597	*Klebsiella pneumoniae*	ESBL

598	*Escherichia coli*	ESBL
599	*Klebsiella pneumoniae*	ESBL
600	*Escherichia coli*	ESBL
601	*Klebsiella pneumoniae*	ESBL
602	*Klebsiella aerogenes*	ESBL
603	*Escherichia coli*	ESBL
604	*Escherichia coli*	ESBL
605	*Enterobacter cloacae complex*	ESBL
606	*Staphylococcus aureus*	MRSA
607	*Staphylococcus aureus*	MRSA
608	*Klebsiella pneumoniae*	ESBL
609	*Staphylococcus aureus*	MRSA
610	*Staphylococcus aureus*	MRSA
611	*Escherichia coli*	ESBL
612	*Escherichia coli*	ESBL
613	*Escherichia coli*	ESBL
614	*Escherichia coli*	ESBL
615	*Klebsiella pneumoniae*	ESBL
616	*Klebsiella pneumoniae*	ESBL
617	*Acinetobacter baumannii*	OXA23
618	*Enterobacter cloacae complex*	ESBL
619	*Klebsiella pneumoniae*	ESBL
620	*Klebsiella pneumoniae*	ESBL
621	*Escherichia coli*	ESBL
622	*Escherichia coli*	ESBL
623	*Klebsiella oxytoca*	ESBL
624	*Escherichia coli*	ESBL
625	*Escherichia coli*	ESBL
626	*Klebsiella pneumoniae*	ESBL
627	*Klebsiella pneumoniae*	OXA48
628	*Klebsiella pneumoniae*	ESBL
629	*Escherichia coli*	ESBL
630	*Klebsiella pneumoniae*	ESBL
631	*Enterobacter cloacae complex*	ESBL
632	*Klebsiella pneumoniae*	ESBL
633	*Escherichia coli*	ESBL
634	*Enterobacter cloacae complex*	ESBL
635	*Klebsiella pneumoniae*	ESBL
636	*Acinetobacter baumannii*	NDM
637	*Enterobacter cloacae complex*	NDM
638	*Escherichia coli*	ESBL
639	*Klebsiella pneumoniae*	ESBL
640	*Enterobacter cloacae complex*	ESBL
641	*Klebsiella pneumoniae*	ESBL
642	*Klebsiella pneumoniae*	ESBL
643	*Escherichia coli*	ESBL

644	*Klebsiella pneumoniae*	NDM, OXA48
645	*Staphylococcus aureus*	MRSA
646	*Escherichia coli*	ESBL
647	*Klebsiella pneumoniae*	ESBL
648	*Escherichia coli*	ESBL
649	*Klebsiella pneumoniae*	ESBL
650	*Klebsiella pneumoniae*	ESBL
651	*Klebsiella pneumoniae*	ESBL
652	*Escherichia coli*	ESBL
653	*Staphylococcus aureus*	MRSA
654	*Klebsiella pneumoniae*	ESBL
655	*Acinetobacter baumannii*	OXA23
656	*Escherichia coli*	ESBL
657	*Klebsiella pneumoniae*	ESBL
658	*Escherichia coli*	ESBL
659	*Klebsiella pneumoniae*	ESBL
660	*Staphylococcus aureus*	MRSA
661	*Escherichia coli*	ESBL
662	*Escherichia coli*	ESBL
663	*Staphylococcus aureus*	MRSA
664	*Klebsiella aerogenes*	ESBL
665	*Staphylococcus aureus*	MRSA
666	*Escherichia coli*	ESBL
667	*Klebsiella pneumoniae*	ESBL
668	*Acinetobacter baumannii*	OXA23
669	*Escherichia coli*	ESBL
670	*Klebsiella pneumoniae*	ESBL
671	*Escherichia coli*	ESBL
672	*Escherichia coli*	ESBL
673	*Escherichia coli*	ESBL
674	*Klebsiella pneumoniae*	ESBL
675	*Escherichia coli*	ESBL
676	*Klebsiella pneumoniae*	ESBL
677	*Klebsiella pneumoniae*	ESBL
678	*Escherichia coli*	ESBL
679	*Escherichia coli*	ESBL
680	*Acinetobacter baumannii*	OXA23
681	*Escherichia coli*	ESBL
682	*Escherichia coli*	ESBL
683	*Enterobacter cloacae complex*	ESBL
684	*Klebsiella pneumoniae*	ESBL
685	*Klebsiella pneumoniae*	ESBL
686	*Enterobacter cloacae complex*	ESBL
687	*Escherichia coli*	ESBL
688	*Escherichia coli*	ESBL
689	*Klebsiella pneumoniae*	ESBL

690	*Klebsiella pneumoniae*	ESBL
691	*Citrobacter freundii*	ESBL
692	*Citrobacter koseri*	ESBL
693	*Staphylococcus aureus*	MRSA
694	*Escherichia coli*	ESBL
695	*Klebsiella pneumoniae*	ESBL
696	*Escherichia coli*	ESBL
697	*Staphylococcus aureus*	MRSA
698	*Klebsiella pneumoniae*	ESBL
699	*Enterobacter cloacae complex*	ESBL
700	*Escherichia coli*	ESBL
701	*Staphylococcus aureus*	MRSA
702	*Escherichia coli*	ESBL
703	*Klebsiella pneumoniae*	ESBL
704	*Staphylococcus aureus*	MRSA
705	*Staphylococcus aureus*	MRSA
706	*Klebsiella pneumoniae*	ESBL
707	*Klebsiella pneumoniae*	ESBL
708	*Escherichia coli*	ESBL
709	*Escherichia coli*	ESBL
710	*Staphylococcus aureus*	MRSA
711	*Enterobacter cloacae complex*	ESBL
712	*Klebsiella pneumoniae*	ESBL
713	*Klebsiella aerogenes*	ESBL
714	*Klebsiella pneumoniae*	ESBL
715	*Staphylococcus aureus*	MRSA
716	*Klebsiella aerogenes*	ESBL
717	*Staphylococcus aureus*	MRSA
718	*Enterobacter cloacae complex*	ESBL
719	*Klebsiella pneumoniae*	ESBL
720	*Escherichia coli*	ESBL
721	*Escherichia coli*	ESBL
722	*Klebsiella pneumoniae*	ESBL
723	*Escherichia coli*	ESBL
724	*Klebsiella pneumoniae*	ESBL
725	*Enterobacter cloacae complex*	VIM
726	*Escherichia coli*	ESBL
727	*Escherichia coli*	ESBL
728	*Citrobacter koseri*	NDM, OXA48
729	*Klebsiella pneumoniae*	NDM, OXA48
730	*Staphylococcus aureus*	MRSA
731	*Escherichia coli*	ESBL
732	*Klebsiella pneumoniae*	ESBL
733	*Escherichia coli*	ESBL
734	*Staphylococcus aureus*	MRSA

735	*Escherichia coli*	ESBL
736	*Klebsiella pneumoniae*	ESBL
737	*Klebsiella aerogenes*	ESBL
738	*Acinetobacter baumannii*	OXA23
739	*Klebsiella pneumoniae*	ESBL
740	*Staphylococcus aureus*	MRSA
741	*Escherichia coli*	ESBL
742	*Escherichia coli*	ESBL
743	*Escherichia coli*	ESBL
744	*Staphylococcus aureus*	MRSA
745	*Staphylococcus aureus*	MRSA
746	*Escherichia coli*	ESBL
747	*Staphylococcus aureus*	MRSA
748	*Escherichia coli*	ESBL
749	*Escherichia coli*	ESBL
750	*Enterobacter cloacae complex*	ESBL
751	*Enterobacter cloacae complex*	ESBL
752	*Escherichia coli*	ESBL
753	*Escherichia coli*	ESBL
754	*Escherichia coli*	ESBL
755	*Klebsiella pneumoniae*	ESBL
756	*Escherichia coli*	ESBL
757	*Klebsiella aerogenes*	ESBL
758	*Citrobacter freundii*	ESBL
759	*Escherichia coli*	ESBL
760	*Klebsiella pneumoniae*	ESBL
761	*Acinetobacter baumannii*	OXA23
762	*Escherichia coli*	ESBL
763	*Klebsiella pneumoniae*	ESBL
764	*Enterobacter cloacae complex*	ESBL
765	*Escherichia coli*	ESBL
766	*Escherichia coli*	ESBL
767	*Staphylococcus aureus*	MRSA
768	*Klebsiella pneumoniae*	ESBL
769	*Escherichia coli*	ESBL
770	*Klebsiella pneumoniae*	ESBL
771	*Escherichia coli*	ESBL
772	*Klebsiella pneumoniae*	ESBL
773	*Escherichia coli*	ESBL
774	*Staphylococcus aureus*	MRSA
775	*Klebsiella pneumoniae*	ESBL
776	*Citrobacter braakii*	ESBL
777	*Escherichia coli*	ESBL
778	*Escherichia coli*	ESBL
779	*Klebsiella pneumoniae*	ESBL
780	*Klebsiella pneumoniae*	ESBL

781	*Klebsiella pneumoniae*	ESBL
782	*Staphylococcus aureus*	MRSA
783	*Enterobacter cloacae complex*	ESBL
784	*Klebsiella pneumoniae*	ESBL
785	*Klebsiella pneumoniae*	ESBL
786	*Escherichia coli*	ESBL
787	*Klebsiella pneumoniae*	ESBL
788	*Klebsiella pneumoniae*	ESBL
789	*Enterobacter cloacae complex*	ESBL
790	*Escherichia coli*	ESBL
791	*Escherichia coli*	ESBL
792	*Enterobacter cloacae complex*	ESBL
793	*Staphylococcus aureus*	MRSA
794	*Staphylococcus aureus*	MRSA
795	*Staphylococcus aureus*	MRSA
796	*Staphylococcus aureus*	MRSA
797	*Enterobacter cloacae complex*	ESBL
798	*Klebsiella pneumoniae*	ESBL
799	*Klebsiella pneumoniae*	ESBL
800	*Enterobacter cloacae complex*	ESBL
801	*Klebsiella pneumoniae*	ESBL
802	*Klebsiella pneumoniae*	ESBL
803	*Escherichia coli*	ESBL
804	*Escherichia coli*	ESBL
805	*Klebsiella pneumoniae*	ESBL
806	*Escherichia coli*	ESBL
807	*Escherichia coli*	ESBL
808	*Escherichia coli*	ESBL
809	*Klebsiella pneumoniae*	ESBL
810	*Escherichia coli*	ESBL
811	*Escherichia coli*	ESBL
812	*Klebsiella pneumoniae*	ESBL
813	*Enterococcus faecium*	VanA
814	*Raoultella planticola*	ESBL
815	*Escherichia coli*	ESBL
816	*Staphylococcus aureus*	MRSA
817	*Staphylococcus aureus*	MRSA
818	*Klebsiella pneumoniae*	ESBL
819	*Enterobacter cloacae complex*	ESBL
820	*Staphylococcus aureus*	MRSA
821	*Staphylococcus aureus*	MRSA
822	*Escherichia coli*	ESBL
823	*Acinetobacter baumannii*	NDM
824	*Enterobacter cloacae complex*	ESBL
825	*Staphylococcus aureus*	MRSA
826	*Klebsiella pneumoniae*	ESBL

827	*Klebsiella pneumoniae*	ESBL
828	*Klebsiella pneumoniae*	ESBL
829	*Klebsiella pneumoniae*	ESBL
830	*Staphylococcus aureus*	MRSA
831	*Enterobacter cloacae complex*	ESBL
832	*Staphylococcus aureus*	MRSA
833	*Escherichia coli*	ESBL
834	*Escherichia coli*	ESBL
835	*Klebsiella pneumoniae*	ESBL
836	*Escherichia coli*	ESBL
837	*Klebsiella pneumoniae*	ESBL
838	*Klebsiella pneumoniae*	ESBL
839	*Enterobacter cloacae complex*	ESBL
840	*Escherichia coli*	ESBL
841	*Escherichia coli*	ESBL
842	*Klebsiella pneumoniae*	ESBL
843	*Klebsiella pneumoniae*	ESBL
844	*Klebsiella pneumoniae*	ESBL
845	*Klebsiella pneumoniae*	ESBL
846	*Klebsiella pneumoniae*	ESBL
847	*Escherichia coli*	ESBL
848	*Staphylococcus aureus*	MRSA
849	*Klebsiella pneumoniae*	ESBL
850	*Escherichia coli*	ESBL
851	*Staphylococcus aureus*	MRSA
852	*Escherichia coli*	ESBL
853	*Enterobacter cloacae complex*	ESBL
854	*Citrobacter koseri*	ESBL
855	*Escherichia coli*	OXA48
856	*Klebsiella pneumoniae*	ESBL
857	*Staphylococcus aureus*	MRSA
858	*Staphylococcus aureus*	MRSA
859	*Klebsiella pneumoniae*	ESBL
860	*Escherichia coli*	ESBL
861	*Enterobacter cloacae complex*	ESBL
862	*Escherichia coli*	ESBL
863	*Escherichia coli*	ESBL
864	*Escherichia coli*	ESBL
865	*Enterobacter cloacae complex*	ESBL
866	*Klebsiella pneumoniae*	ESBL
867	*Escherichia coli*	ESBL
868	*Staphylococcus aureus*	MRSA
869	*Escherichia coli*	ESBL
870	*Escherichia coli*	ESBL
871	*Escherichia coli*	ESBL
872	*Escherichia coli*	ESBL

873	*Escherichia coli*	ESBL
874	*Escherichia coli*	ESBL
875	*Escherichia coli*	ESBL
876	*Klebsiella pneumoniae*	ESBL
877	*Enterobacter cloacae complex*	ESBL
878	*Klebsiella pneumoniae*	ESBL
879	*Escherichia coli*	ESBL
880	*Escherichia coli*	ESBL
881	*Klebsiella pneumoniae*	ESBL
882	*Staphylococcus aureus*	MRSA
883	*Escherichia coli*	ESBL
884	*Escherichia coli*	ESBL
885	*Klebsiella pneumoniae*	ESBL
886	*Enterobacter cloacae complex*	ESBL
887	*Staphylococcus aureus*	MRSA
888	*Klebsiella pneumoniae*	ESBL
889	*Citrobacter freundii*	ESBL
890	*Acinetobacter baumannii*	OXA23
891	*Klebsiella pneumoniae*	ESBL
892	*Escherichia coli*	ESBL
893	*Klebsiella pneumoniae*	ESBL
894	*Klebsiella pneumoniae*	ESBL
895	*Escherichia coli*	OXA48
896	*Klebsiella pneumoniae*	OXA48
897	*Citrobacter koseri*	ESBL
898	*Enterobacter cloacae complex*	ESBL
899	*Escherichia coli*	ESBL
900	*Klebsiella pneumoniae*	ESBL
901	*Klebsiella pneumoniae*	ESBL
902	*Klebsiella pneumoniae*	ESBL
903	*Klebsiella oxytoca*	ESBL
904	*Escherichia coli*	ESBL
905	*Klebsiella pneumoniae*	ESBL
906	*Escherichia coli*	ESBL
907	*Acinetobacter baumannii*	OXA23
908	*Escherichia coli*	ESBL
909	*Enterobacter cloacae complex*	VIM
910	*Staphylococcus aureus*	MRSA
911	*Escherichia coli*	ESBL
912	*Escherichia coli*	ESBL
913	*Klebsiella pneumoniae*	ESBL
914	*Klebsiella pneumoniae*	ESBL
915	*Escherichia coli*	ESBL
916	*Escherichia coli*	ESBL
917	*Staphylococcus aureus*	MRSA
918	*Klebsiella pneumoniae*	ESBL

919	*Escherichia coli*	ESBL
920	*Escherichia coli*	ESBL
921	*Enterobacter cloacae complex*	ESBL
922	*Escherichia coli*	ESBL
923	*Citrobacter freundii*	ESBL
924	*Klebsiella pneumoniae*	ESBL
925	*Klebsiella oxytoca*	ESBL
926	*Klebsiella pneumoniae*	ESBL
927	*Klebsiella pneumoniae*	ESBL
928	*Klebsiella pneumoniae*	ESBL
929	*Klebsiella pneumoniae*	ESBL
930	*Staphylococcus aureus*	MRSA
931	*Escherichia coli*	ESBL
932	*Escherichia coli*	ESBL
933	*Klebsiella pneumoniae*	ESBL
934	*Klebsiella pneumoniae*	ESBL
935	*Klebsiella pneumoniae*	ESBL
936	*Escherichia coli*	ESBL
937	*Enterobacter cloacae complex*	ESBL
938	*Klebsiella pneumoniae*	ESBL
939	*Escherichia coli*	ESBL
940	*Escherichia coli*	ESBL
941	*Escherichia coli*	ESBL
942	*Escherichia coli*	ESBL
943	*Enterobacter cloacae complex*	ESBL
944	*Escherichia coli*	ESBL
945	*Klebsiella pneumoniae*	ESBL
946	*Escherichia coli*	ESBL
947	*Escherichia coli*	ESBL
948	*Staphylococcus aureus*	MRSA
949	*Klebsiella pneumoniae*	ESBL
950	*Escherichia coli*	ESBL
951	*Escherichia coli*	ESBL
952	*Enterobacter cloacae complex*	ESBL
953	*Escherichia coli*	OXA48
954	*Enterobacter cloacae complex*	OXA48
955	*Klebsiella oxytoca*	ESBL
956	*Staphylococcus aureus*	MRSA
957	*Escherichia coli*	ESBL
958	*Escherichia coli*	ESBL
959	*Escherichia coli*	ESBL
960	*Klebsiella pneumoniae*	ESBL
961	*Escherichia coli*	ESBL
962	*Enterobacter cloacae complex*	ESBL
963	*Escherichia coli*	ESBL
964	*Enterobacter cloacae complex*	ESBL

965	*Klebsiella pneumoniae*	ESBL
966	*Escherichia coli*	ESBL
967	*Enterobacter cloacae complex*	ESBL
968	*Klebsiella pneumoniae*	ESBL
969	*Klebsiella pneumoniae*	ESBL
970	*Enterobacter cloacae complex*	ESBL
971	*Staphylococcus aureus*	MRSA
972	*Escherichia coli*	ESBL
973	*Escherichia coli*	ESBL
974	*Escherichia coli*	ESBL
975	*Escherichia coli*	ESBL
976	*Staphylococcus aureus*	MRSA
977	*Escherichia coli*	ESBL
978	*Staphylococcus aureus*	MRSA
979	*Staphylococcus aureus*	MRSA
980	*Escherichia coli*	ESBL
981	*Escherichia coli*	ESBL
982	*Klebsiella pneumoniae*	ESBL
983	*Klebsiella pneumoniae*	ESBL
984	*Staphylococcus aureus*	MRSA
985	*Klebsiella pneumoniae*	ESBL
986	*Escherichia coli*	ESBL
987	*Klebsiella pneumoniae*	ESBL
988	*Staphylococcus aureus*	MRSA
989	*Klebsiella pneumoniae*	ESBL
990	*Staphylococcus aureus*	MRSA
991	*Staphylococcus aureus*	MRSA
992	*Klebsiella pneumoniae*	ESBL
993	*Klebsiella pneumoniae*	ESBL
994	*Enterobacter cloacae complex*	ESBL
995	*Escherichia coli*	ESBL
996	*Enterobacter cloacae complex*	ESBL
997	*Escherichia coli*	ESBL
998	*Staphylococcus aureus*	MRSA
999	*Escherichia coli*	ESBL
1000	*Klebsiella pneumoniae*	ESBL
1001	*Escherichia coli*	ESBL
1002	*Escherichia coli*	ESBL
1003	*Escherichia coli*	ESBL
1004	*Citrobacter freundii*	ESBL
1005	*Acinetobacter baumannii*	OXA23
1006	*Klebsiella pneumoniae*	ESBL
1007	*Enterobacter cloacae complex*	ESBL
1008	*Staphylococcus aureus*	MRSA
1009	*Staphylococcus aureus*	MRSA
1010	*Klebsiella pneumoniae*	ESBL

1011	*Enterobacter cloacae complex*	VIM
1012	*Escherichia coli*	ESBL
1013	*Staphylococcus aureus*	MRSA
1014	*Enterobacter cloacae complex*	ESBL
1015	*Klebsiella pneumoniae*	ESBL
1016	*Klebsiella pneumoniae*	ESBL
1017	*Klebsiella pneumoniae*	ESBL
1018	*Acinetobacter baumannii*	NDM
1019	*Klebsiella pneumoniae*	ESBL
1020	*Escherichia coli*	ESBL
1021	*Klebsiella pneumoniae*	ESBL
1022	*Enterobacter cloacae complex*	ESBL
1023	*Klebsiella pneumoniae*	ESBL
1024	*Enterobacter cloacae complex*	ESBL
1025	*Escherichia coli*	ESBL
1026	*Escherichia coli*	ESBL
1027	*Klebsiella pneumoniae*	ESBL
1028	*Escherichia coli*	ESBL
1029	*Klebsiella pneumoniae*	ESBL
1030	*Klebsiella pneumoniae*	ESBL
1031	*Escherichia coli*	ESBL
1032	*Escherichia coli*	ESBL
1033	*Enterobacter cloacae complex*	VIM
1034	*Escherichia coli*	ESBL
1035	*Klebsiella pneumoniae*	ESBL
1036	*Escherichia coli*	ESBL
1037	*Escherichia coli*	ESBL
1038	*Klebsiella pneumoniae*	ESBL
1039	*Klebsiella pneumoniae*	ESBL
1040	*Acinetobacter baumannii*	OXA23
1041	*Escherichia coli*	ESBL
1042	*Staphylococcus aureus*	MRSA
1043	*Escherichia coli*	ESBL
1044	*Klebsiella pneumoniae*	ESBL
1045	*Escherichia coli*	ESBL
1046	*Klebsiella pneumoniae*	ESBL
1047	*Escherichia coli*	ESBL
1048	*Staphylococcus aureus*	MRSA
1049	*Escherichia coli*	ESBL
1050	*Staphylococcus aureus*	MRSA
1051	*Klebsiella pneumoniae*	NDM, OXA48
1052	*Acinetobacter baumannii*	OXA23
1053	*Citrobacter farmeri*	ESBL
1054	*Klebsiella oxytoca*	ESBL
1055	*Escherichia coli*	ESBL
1056	*Klebsiella pneumoniae*	ESBL

1057	*Escherichia coli*	ESBL
1058	*Citrobacter freundii*	ESBL
1059	*Escherichia coli*	ESBL
1060	*Enterobacter cloacae complex*	ESBL
1061	*Escherichia coli*	ESBL
1062	*Enterobacter cloacae complex*	ESBL
1063	*Escherichia coli*	ESBL
1064	*Klebsiella pneumoniae*	ESBL
1065	*Escherichia coli*	ESBL
1066	*Escherichia coli*	ESBL
1067	*Klebsiella pneumoniae*	ESBL
1068	*Klebsiella pneumoniae*	ESBL
1069	*Klebsiella pneumoniae*	ESBL
1070	*Klebsiella pneumoniae*	ESBL
1071	*Escherichia coli*	OXA48
1072	*Klebsiella pneumoniae*	ESBL
1073	*Klebsiella pneumoniae*	ESBL
1074	*Escherichia coli*	ESBL
1075	*Enterobacter cloacae complex*	ESBL
1076	*Staphylococcus aureus*	MRSA
1077	*Escherichia coli*	ESBL
1078	*Klebsiella pneumoniae*	ESBL
1079	*Citrobacter freundii*	NDM
1080	*Klebsiella pneumoniae*	ESBL
1081	*Escherichia coli*	ESBL
1082	*Klebsiella pneumoniae*	ESBL
1083	*Staphylococcus aureus*	MRSA
1084	*Escherichia coli*	ESBL
1085	*Escherichia coli*	ESBL
1086	*Klebsiella pneumoniae*	ESBL
1087	*Escherichia coli*	ESBL
1088	*Escherichia coli*	ESBL
1089	*Escherichia coli*	ESBL
1090	*Klebsiella pneumoniae*	ESBL
1091	*Staphylococcus aureus*	MRSA
1092	*Klebsiella pneumoniae*	ESBL
1093	*Klebsiella pneumoniae*	ESBL
1094	*Klebsiella pneumoniae*	ESBL
1095	*Enterobacter cloacae complex*	ESBL
1096	*Escherichia coli*	ESBL
1097	*Staphylococcus aureus*	MRSA
1098	*Klebsiella pneumoniae*	ESBL
1099	*Escherichia coli*	ESBL
1100	*Escherichia coli*	ESBL
1101	*Staphylococcus aureus*	MRSA
1102	*Escherichia coli*	ESBL

1103	*Enterobacter cloacae complex*	ESBL
1104	*Escherichia coli*	ESBL
1105	*Klebsiella pneumoniae*	ESBL
1106	*Klebsiella pneumoniae*	ESBL
1107	*Klebsiella pneumoniae*	ESBL
1108	*Enterobacter cloacae complex*	VIM
1109	*Klebsiella pneumoniae*	ESBL
1110	*Staphylococcus aureus*	MRSA
1111	*Klebsiella pneumoniae*	ESBL
1112	*Enterobacter cloacae complex*	ESBL
1113	*Escherichia coli*	ESBL
1114	*Klebsiella pneumoniae*	ESBL
1115	*Escherichia coli*	ESBL
1116	*Escherichia coli*	ESBL
1117	*Escherichia coli*	ESBL
1118	*Klebsiella pneumoniae*	ESBL
1119	*Klebsiella pneumoniae*	ESBL
1120	*Escherichia coli*	ESBL
1121	*Escherichia coli*	ESBL
1122	*Klebsiella pneumoniae*	ESBL
1123	*Escherichia coli*	ESBL
1124	*Escherichia coli*	ESBL
1125	*Acinetobacter baumannii*	IMP
1126	*Klebsiella pneumoniae*	IMP
1127	*Escherichia coli*	ESBL
1128	*Klebsiella pneumoniae*	ESBL
1129	*Staphylococcus aureus*	MRSA
1130	*Escherichia coli*	ESBL
1131	*Klebsiella pneumoniae*	ESBL
1132	*Enterobacter cloacae complex*	ESBL
1133	*Enterobacter cloacae complex*	ESBL
1134	*Escherichia coli*	ESBL
1135	*Escherichia coli*	ESBL
1136	*Escherichia coli*	ESBL
1137	*Klebsiella pneumoniae*	ESBL
1138	*Klebsiella pneumoniae*	ESBL
1139	*Escherichia coli*	ESBL
1140	*Klebsiella pneumoniae*	ESBL
1141	*Enterobacter cloacae complex*	ESBL
1142	*Escherichia coli*	ESBL
1143	*Escherichia coli*	ESBL
1144	*Staphylococcus aureus*	MRSA
1145	*Klebsiella pneumoniae*	ESBL
1146	*Acinetobacter baumannii*	OXA23
1147	*Klebsiella pneumoniae*	ESBL
1148	*Staphylococcus aureus*	MRSA

1149	*Klebsiella pneumoniae*	ESBL
1150	*Enterobacter cloacae complex*	ESBL
1151	*Klebsiella pneumoniae*	ESBL
1152	*Acinetobacter baumannii*	OXA23
1153	*Escherichia coli*	ESBL
1154	*Klebsiella pneumoniae*	ESBL
1155	*Escherichia coli*	ESBL
1156	*Escherichia coli*	ESBL
1157	*Escherichia coli*	ESBL
1158	*Staphylococcus aureus*	MRSA
1159	*Staphylococcus aureus*	MRSA
1160	*Acinetobacter baumannii*	NDM
1161	*Enterobacter cloacae complex*	NDM
1162	*Escherichia coli*	ESBL
1163	*Escherichia coli*	ESBL
1164	*Enterobacter cloacae complex*	ESBL
1165	*Escherichia coli*	ESBL
1166	*Acinetobacter baumannii*	OXA23
1167	*Klebsiella pneumoniae*	ESBL
1168	*Escherichia coli*	ESBL
1169	*Staphylococcus aureus*	MRSA
1170	*Klebsiella pneumoniae*	ESBL
1171	*Citrobacter freundii*	ESBL
1172	*Klebsiella pneumoniae*	ESBL
1173	*Klebsiella pneumoniae*	ESBL
1174	*Escherichia coli*	ESBL
1175	*Klebsiella pneumoniae*	ESBL
1176	*Escherichia coli*	ESBL
1177	*Enterobacter cloacae complex*	ESBL
1178	*Escherichia coli*	ESBL
1179	*Klebsiella pneumoniae*	ESBL
1180	*Escherichia coli*	ESBL
1181	*Klebsiella pneumoniae*	ESBL
1182	*Escherichia coli*	ESBL
1183	*Klebsiella pneumoniae*	ESBL
1184	*Klebsiella pneumoniae*	ESBL
1185	*Staphylococcus aureus*	MRSA
1186	*Escherichia coli*	ESBL
1187	*Escherichia coli*	ESBL
1188	*Klebsiella pneumoniae*	ESBL
1189	*Enterobacter cloacae complex*	ESBL
1190	*Escherichia coli*	ESBL
1191	*Escherichia coli*	ESBL
1192	*Klebsiella pneumoniae*	ESBL
1193	*Escherichia coli*	ESBL
1194	*Staphylococcus aureus*	MRSA

1195	*Staphylococcus aureus*	MRSA
1196	*Klebsiella pneumoniae*	OXA48
1197	*Escherichia coli*	ESBL
1198	*Staphylococcus aureus*	MRSA
1199	*Escherichia coli*	ESBL
1200	*Klebsiella pneumoniae*	ESBL
1201	*Escherichia coli*	ESBL
1202	*Staphylococcus aureus*	MRSA
1203	*Klebsiella pneumoniae*	ESBL
1204	*Escherichia coli*	ESBL
1205	*Escherichia coli*	ESBL
1206	*Staphylococcus aureus*	MRSA
1207	*Staphylococcus aureus*	MRSA
1208	*Escherichia coli*	ESBL
1209	*Klebsiella pneumoniae*	ESBL
1210	*Escherichia coli*	ESBL
1211	*Escherichia coli*	ESBL
1212	*Staphylococcus aureus*	MRSA
1213	*Klebsiella pneumoniae*	ESBL
1214	*Escherichia coli*	ESBL
1215	*Klebsiella pneumoniae*	ESBL
1216	*Acinetobacter baumannii*	OXA23
1217	*Staphylococcus aureus*	MRSA
1218	*Enterobacter cloacae complex*	ESBL
1219	*Escherichia coli*	ESBL
1220	*Klebsiella pneumoniae*	ESBL
1221	*Acinetobacter baumannii*	OXA23
1222	*Escherichia coli*	ESBL
1223	*Escherichia coli*	ESBL
1224	*Klebsiella pneumoniae*	ESBL
1225	*Enterobacter cloacae complex*	ESBL
1226	*Escherichia coli*	ESBL
1227	*Klebsiella pneumoniae*	ESBL
1228	*Klebsiella pneumoniae*	ESBL
1229	*Escherichia coli*	ESBL
1230	*Escherichia coli*	ESBL
1231	*Klebsiella pneumoniae*	ESBL
1232	*Enterobacter cloacae complex*	ESBL
1233	*Escherichia coli*	ESBL
1234	*Staphylococcus aureus*	MRSA
1235	*Klebsiella pneumoniae*	ESBL
1236	*Escherichia coli*	ESBL
1237	*Escherichia coli*	ESBL
1238	*Klebsiella pneumoniae*	ESBL
1239	*Enterobacter cloacae complex*	ESBL
1240	*Enterobacter cloacae complex*	ESBL

1241	*Klebsiella pneumoniae*	ESBL
1242	*Klebsiella pneumoniae*	ESBL
1243	*Klebsiella pneumoniae*	ESBL
1244	*Escherichia coli*	ESBL
1245	*Escherichia coli*	ESBL
1246	*Klebsiella pneumoniae*	ESBL
1247	*Escherichia coli*	ESBL
1248	*Citrobacter koseri*	ESBL
1249	*Acinetobacter baumannii*	OXA23
1250	*Staphylococcus aureus*	MRSA
1251	*Escherichia coli*	ESBL
1252	*Escherichia coli*	ESBL
1253	*Klebsiella pneumoniae*	ESBL
1254	*Klebsiella pneumoniae*	ESBL
1255	*Escherichia coli*	ESBL
1256	*Klebsiella pneumoniae*	ESBL
1257	*Acinetobacter baumannii*	NDM
1258	*Escherichia coli*	ESBL
1259	*Klebsiella pneumoniae*	ESBL
1260	*Klebsiella pneumoniae*	ESBL
1261	*Escherichia coli*	ESBL
1262	*Staphylococcus aureus*	MRSA
1263	*Klebsiella pneumoniae*	ESBL
1264	*Enterobacter cloacae complex*	ESBL
1265	*Staphylococcus aureus*	MRSA
1266	*Klebsiella pneumoniae*	ESBL
1267	*Escherichia coli*	ESBL
1268	*Escherichia coli*	ESBL
1269	*Klebsiella pneumoniae*	ESBL
1270	*Klebsiella pneumoniae*	ESBL
1271	*Staphylococcus aureus*	MRSA
1272	*Staphylococcus aureus*	MRSA
1273	*Staphylococcus aureus*	MRSA
1274	*Escherichia coli*	ESBL
1275	*Klebsiella variicola*	ESBL
1276	*Escherichia coli*	OXA48
1277	*Klebsiella pneumoniae*	ESBL
1278	*Staphylococcus aureus*	MRSA
1279	*Staphylococcus aureus*	MRSA
1280	*Enterobacter cloacae complex*	ESBL
1281	*Escherichia coli*	ESBL
1282	*Enterobacter cloacae complex*	ESBL
1283	*Citrobacter freundii*	OXA48
1284	*Staphylococcus aureus*	MRSA
1285	*Staphylococcus aureus*	MRSA
1286	*Escherichia coli*	ESBL

1287	*Staphylococcus aureus*	MRSA
1288	*Escherichia coli*	ESBL
1289	*Enterobacter cloacae complex*	ESBL
1290	*Escherichia coli*	ESBL
1291	*Enterobacter cloacae complex*	VIM
1292	*Klebsiella pneumoniae*	ESBL
1293	*Escherichia coli*	ESBL
1294	*Citrobacter freundii*	ESBL
1295	*Staphylococcus aureus*	MRSA
1296	*Enterobacter cloacae complex*	ESBL
1297	*Klebsiella pneumoniae*	ESBL
1298	*Klebsiella pneumoniae*	ESBL
1299	*Escherichia coli*	ESBL
1300	*Klebsiella pneumoniae*	ESBL
1301	*Escherichia coli*	ESBL
1302	*Escherichia coli*	ESBL
1303	*Klebsiella pneumoniae*	ESBL
1304	*Staphylococcus aureus*	MRSA
1305	*Escherichia coli*	ESBL
1306	*Staphylococcus aureus*	MRSA
1307	*Klebsiella pneumoniae*	ESBL
1308	*Escherichia coli*	ESBL
1309	*Enterobacter cloacae complex*	ESBL
1310	*Escherichia coli*	ESBL
1311	*Klebsiella pneumoniae*	ESBL
1312	*Enterobacter cloacae complex*	ESBL
1313	*Klebsiella pneumoniae*	ESBL
1314	*Escherichia coli*	ESBL
1315	*Escherichia coli*	ESBL
1316	*Klebsiella pneumoniae*	ESBL
1317	*Staphylococcus aureus*	MRSA
1318	*Escherichia coli*	ESBL
1319	*Klebsiella pneumoniae*	ESBL
1320	*Escherichia coli*	ESBL
1321	*Klebsiella pneumoniae*	ESBL
1322	*Escherichia coli*	ESBL
1323	*Klebsiella pneumoniae*	ESBL
1324	*Escherichia coli*	ESBL
1325	*Klebsiella pneumoniae*	ESBL
1326	*Staphylococcus aureus*	MRSA
1327	*Staphylococcus aureus*	MRSA
1328	*Klebsiella pneumoniae*	ESBL
1329	*Klebsiella pneumoniae*	ESBL
1330	*Klebsiella pneumoniae*	ESBL
1331	*Citrobacter braakii*	ESBL
1332	*Klebsiella aerogenes*	ESBL

1333	*Klebsiella pneumoniae*	ESBL
1334	*Escherichia coli*	ESBL
1335	*Enterobacter cloacae complex*	ESBL
1336	*Citrobacter amalonaticus*	ESBL
1337	*Escherichia coli*	ESBL
1338	*Klebsiella pneumoniae*	ESBL
1339	*Escherichia coli*	ESBL
1340	*Enterobacter cloacae complex*	ESBL
1341	*Escherichia coli*	ESBL
1342	*Klebsiella pneumoniae*	ESBL
1343	*Staphylococcus aureus*	MRSA
1344	*Staphylococcus aureus*	MRSA
1345	*Escherichia coli*	ESBL
1346	*Klebsiella pneumoniae*	ESBL
1347	*Escherichia coli*	ESBL
1348	*Escherichia coli*	ESBL
1349	*Klebsiella pneumoniae*	ESBL
1350	*Staphylococcus aureus*	MRSA
1351	*Acinetobacter baumannii*	OXA23
1352	*Klebsiella pneumoniae*	ESBL
1353	*Escherichia coli*	ESBL
1354	*Klebsiella pneumoniae*	ESBL
1355	*Staphylococcus aureus*	MRSA
1356	*Escherichia coli*	ESBL
1357	*Escherichia coli*	ESBL
1358	*Staphylococcus aureus*	MRSA
1359	*Klebsiella pneumoniae*	ESBL
1360	*Klebsiella pneumoniae*	ESBL
1361	*Klebsiella pneumoniae*	ESBL
1362	*Escherichia coli*	ESBL
1363	*Klebsiella pneumoniae*	ESBL
1364	*Enterobacter cloacae complex*	ESBL
1365	*Klebsiella pneumoniae*	ESBL
1366	*Klebsiella pneumoniae*	ESBL
1367	*Staphylococcus aureus*	MRSA
1368	*Staphylococcus aureus*	MRSA
1369	*Enterobacter cloacae complex*	ESBL
1370	*Enterobacter cloacae complex*	ESBL
1371	*Escherichia coli*	ESBL
1372	*Klebsiella pneumoniae*	ESBL
1373	*Klebsiella pneumoniae*	ESBL
1374	*Acinetobacter baumannii*	OXA23
1375	*Klebsiella oxytoca*	ESBL
1376	*Citrobacter braakii*	ESBL
1377	*Klebsiella pneumoniae*	ESBL
1378	*Escherichia coli*	ESBL

1379	*Klebsiella pneumoniae*	ESBL
1380	*Klebsiella pneumoniae*	ESBL
1381	*Staphylococcus aureus*	MRSA
1382	*Klebsiella pneumoniae*	ESBL
1383	*Escherichia coli*	ESBL
1384	*Klebsiella pneumoniae*	ESBL
1385	*Klebsiella pneumoniae*	ESBL
1386	*Klebsiella pneumoniae*	ESBL
1387	*Klebsiella pneumoniae*	ESBL
1388	*Escherichia coli*	ESBL
1389	*Enterobacter cloacae complex*	ESBL
1390	*Escherichia coli*	ESBL
1391	*Klebsiella pneumoniae*	ESBL
1392	*Klebsiella pneumoniae*	ESBL
1393	*Escherichia coli*	ESBL
1394	*Enterobacter cloacae complex*	ESBL
1395	*Enterobacter cloacae complex*	ESBL
1396	*Escherichia coli*	ESBL
1397	*Enterobacter cloacae complex*	ESBL
1398	*Escherichia coli*	ESBL
1399	*Klebsiella pneumoniae*	ESBL
1400	*Escherichia coli*	ESBL
1401	*Escherichia coli*	ESBL
1402	*Klebsiella pneumoniae*	ESBL
1403	*Escherichia coli*	ESBL
1404	*Enterobacter cloacae complex*	ESBL
1405	*Escherichia coli*	ESBL
1406	*Klebsiella pneumoniae*	ESBL
1407	*Escherichia coli*	ESBL
1408	*Citrobacter freundii*	ESBL
1409	*Staphylococcus aureus*	MRSA
1410	*Escherichia coli*	ESBL
1411	*Klebsiella pneumoniae*	ESBL
1412	*Escherichia coli*	ESBL
1413	*Klebsiella pneumoniae*	ESBL
1414	*Escherichia coli*	ESBL
1415	*Klebsiella pneumoniae*	ESBL
1416	*Klebsiella pneumoniae*	ESBL
1417	*Escherichia coli*	ESBL
1418	*Staphylococcus aureus*	MRSA
1419	*Acinetobacter baumannii*	OXA23
1420	*Klebsiella pneumoniae*	ESBL
1421	*Enterobacter cloacae complex*	ESBL
1422	*Enterobacter cloacae complex*	ESBL
1423	*Staphylococcus aureus*	MRSA
1424	*Escherichia coli*	ESBL

1425	*Klebsiella pneumoniae*	ESBL
1426	*Klebsiella pneumoniae*	ESBL
1427	*Escherichia coli*	ESBL
1428	*Escherichia coli*	ESBL
1429	*Enterobacter cloacae complex*	ESBL
1430	*Klebsiella pneumoniae*	ESBL
1431	*Escherichia coli*	ESBL
1432	*Escherichia coli*	ESBL
1433	*Citrobacter koseri*	ESBL
1434	*Klebsiella pneumoniae*	ESBL
1435	*Escherichia coli*	ESBL
1436	*Pseudomonas aeruginosa*	ESBL
1437	*Klebsiella pneumoniae*	ESBL
1438	*Escherichia coli*	ESBL
1439	*Klebsiella pneumoniae*	ESBL
1440	*Escherichia coli*	ESBL
1441	*Staphylococcus aureus*	MRSA
1442	*Klebsiella pneumoniae*	ESBL
1443	*Enterobacter cloacae complex*	ESBL
1444	*Escherichia coli*	ESBL
1445	*Escherichia coli*	ESBL
1446	*Escherichia coli*	ESBL
1447	*Escherichia coli*	ESBL
1448	*Escherichia coli*	ESBL
1449	*Escherichia coli*	ESBL
1450	*Klebsiella pneumoniae*	ESBL
1451	*Staphylococcus aureus*	MRSA
1452	*Staphylococcus aureus*	MRSA
1453	*Klebsiella pneumoniae*	ESBL
1454	*Escherichia coli*	ESBL
1455	*Escherichia coli*	ESBL
1456	*Staphylococcus aureus*	MRSA
1457	*Escherichia coli*	ESBL
1458	*Acinetobacter baumannii*	NDM
1459	*Citrobacter koseri*	ESBL
1460	*Escherichia coli*	ESBL
1461	*Escherichia coli*	ESBL
1462	*Staphylococcus aureus*	MRSA
1463	*Enterobacter cloacae complex*	ESBL
1464	*Klebsiella pneumoniae*	ESBL
1465	*Escherichia coli*	ESBL
1466	*Klebsiella pneumoniae*	ESBL
1467	*Staphylococcus aureus*	MRSA
1468	*Klebsiella pneumoniae*	ESBL
1469	*Klebsiella pneumoniae*	ESBL
1470	*Klebsiella pneumoniae*	ESBL

1471	*Klebsiella pneumoniae*	ESBL
1472	*Escherichia coli*	ESBL
1473	*Staphylococcus aureus*	MRSA
1474	*Enterobacter cloacae complex*	VIM
1475	*Escherichia coli*	VIM
1476	*Citrobacter freundii*	ESBL
1477	*Escherichia coli*	ESBL
1478	*Escherichia coli*	ESBL
1479	*Acinetobacter baumannii*	OXA23
1480	*Escherichia coli*	ESBL
1481	*Klebsiella pneumoniae*	ESBL
1482	*Klebsiella pneumoniae*	ESBL
1483	*Escherichia coli*	ESBL
1484	*Enterobacter cloacae complex*	ESBL
1485	*Klebsiella pneumoniae*	ESBL
1486	*Staphylococcus aureus*	MRSA
1487	*Klebsiella pneumoniae*	ESBL
1488	*Escherichia coli*	ESBL
1489	*Klebsiella pneumoniae*	ESBL
1490	*Staphylococcus aureus*	MRSA
1491	*Enterobacter cloacae complex*	ESBL
1492	*Klebsiella pneumoniae*	ESBL
1493	*Pantoea sp*	ESBL
1494	*Klebsiella pneumoniae*	ESBL
1495	*Acinetobacter baumannii*	OXA23
1496	*Klebsiella pneumoniae*	ESBL
1497	*Escherichia coli*	ESBL
1498	*Staphylococcus aureus*	MRSA
1499	*Escherichia coli*	ESBL
1500	*Klebsiella pneumoniae*	ESBL
1501	*Escherichia coli*	ESBL
1502	*Klebsiella pneumoniae*	ESBL
1503	*Klebsiella pneumoniae*	ESBL
1504	*Klebsiella pneumoniae*	ESBL
1505	*Staphylococcus aureus*	MRSA
1506	*Klebsiella pneumoniae*	ESBL
1507	*Escherichia coli*	ESBL
1508	*Escherichia coli*	ESBL
1509	*Staphylococcus aureus*	MRSA
1510	*Klebsiella pneumoniae*	OXA48
1511	*Escherichia coli*	ESBL
1512	*Klebsiella pneumoniae*	ESBL
1513	*Klebsiella pneumoniae*	ESBL
1514	*Acinetobacter baumannii*	OXA23
1515	*Acinetobacter baumannii*	OXA23
1516	*Klebsiella pneumoniae*	ESBL

1517	*Escherichia coli*	ESBL
1518	*Klebsiella pneumoniae*	ESBL
1519	*Escherichia coli*	ESBL
1520	*Klebsiella pneumoniae*	ESBL
1521	*Escherichia coli*	ESBL
1522	*Escherichia coli*	ESBL
1523	*Escherichia coli*	ESBL
1524	*Klebsiella pneumoniae*	ESBL
1525	*Escherichia coli*	ESBL
1526	*Staphylococcus aureus*	MRSA
1527	*Enterobacter cloacae complex*	ESBL
1528	*Escherichia coli*	ESBL
1529	*Klebsiella pneumoniae*	ESBL
1530	*Escherichia coli*	ESBL
1531	*Escherichia coli*	ESBL
1532	*Klebsiella pneumoniae*	ESBL
1533	*Escherichia coli*	OXA48
1534	*Escherichia coli*	ESBL
1535	*Escherichia coli*	ESBL
1536	*Klebsiella pneumoniae*	ESBL
1537	*Escherichia coli*	ESBL
1538	*Enterobacter cloacae complex*	ESBL
1539	*Klebsiella pneumoniae*	ESBL
1540	*Enterobacter cloacae complex*	ESBL
1541	*Klebsiella pneumoniae*	ESBL
1542	*Klebsiella pneumoniae*	ESBL
1543	*Escherichia coli*	ESBL
1544	*Klebsiella pneumoniae*	ESBL
1545	*Escherichia coli*	ESBL
1546	*Klebsiella pneumoniae*	ESBL
1547	*Enterobacter cloacae complex*	ESBL
1548	*Klebsiella pneumoniae*	ESBL
1549	*Enterobacter cloacae complex*	ESBL
1550	*Staphylococcus aureus*	MRSA
1551	*Enterobacter cloacae complex*	ESBL
1552	*Klebsiella pneumoniae*	ESBL
1553	*Staphylococcus aureus*	MRSA
1554	*Klebsiella pneumoniae*	ESBL
1555	*Escherichia coli*	ESBL
1556	*Citrobacter koseri*	ESBL
1557	*Klebsiella pneumoniae*	ESBL
1558	*Klebsiella oxytoca*	ESBL
1559	*Escherichia coli*	ESBL
1560	*Staphylococcus aureus*	MRSA
1561	*Staphylococcus aureus*	MRSA
1562	*Klebsiella pneumoniae*	ESBL

1563	*Acinetobacter baumannii*	OXA23
1564	*Escherichia coli*	ESBL
1565	*Escherichia coli*	ESBL
1566	*Klebsiella pneumoniae*	ESBL
1567	*Escherichia coli*	ESBL
1568	*Citrobacter freundii*	ESBL
1569	*Enterobacter cloacae complex*	VIM
1570	*Klebsiella pneumoniae*	ESBL
1571	*Staphylococcus aureus*	MRSA
1572	*Escherichia coli*	ESBL
1573	*Citrobacter koseri*	ESBL
1574	*Escherichia coli*	ESBL
1575	*Klebsiella pneumoniae*	ESBL
1576	*Klebsiella pneumoniae*	ESBL
1577	*Enterobacter cloacae complex*	ESBL
1578	*Escherichia coli*	ESBL
1579	*Escherichia coli*	ESBL
1580	*Escherichia coli*	ESBL
1581	*Klebsiella pneumoniae*	ESBL
1582	*Klebsiella pneumoniae*	ESBL
1583	*Klebsiella pneumoniae*	ESBL
1584	*Morganella morganii*	ESBL
1585	*Enterobacter cloacae complex*	ESBL
1586	*Klebsiella pneumoniae*	ESBL
1587	*Escherichia coli*	ESBL
1588	*Escherichia coli*	ESBL
1589	*Klebsiella pneumoniae*	ESBL
1590	*Escherichia coli*	ESBL
1591	*Escherichia coli*	ESBL
1592	*Staphylococcus aureus*	MRSA
1593	*Escherichia coli*	ESBL
1594	*Klebsiella aerogenes*	ESBL
1595	*Klebsiella pneumoniae*	ESBL
1596	*Escherichia coli*	ESBL
1597	*Klebsiella pneumoniae*	ESBL
1598	*Escherichia coli*	ESBL
1599	*Klebsiella pneumoniae*	ESBL
1600	*Escherichia coli*	ESBL
1601	*Staphylococcus aureus*	MRSA
1602	*Escherichia coli*	OXA48
1603	*Escherichia coli*	ESBL
1604	*Escherichia coli*	ESBL
1605	*Escherichia coli*	ESBL
1606	*Escherichia coli*	ESBL
1607	*Escherichia coli*	ESBL
1608	*Escherichia coli*	ESBL

1609	*Staphylococcus aureus*	MRSA
1610	*Citrobacter freundii*	ESBL
1611	*Escherichia coli*	OXA48
1612	*Klebsiella oxytoca*	OXA48
1613	*Escherichia coli*	ESBL
1614	*Escherichia coli*	ESBL
1615	*Escherichia coli*	ESBL
1616	*Escherichia coli*	ESBL
1617	*Klebsiella pneumoniae*	ESBL
1618	*Klebsiella pneumoniae*	ESBL
1619	*Escherichia coli*	ESBL
1620	*Escherichia coli*	ESBL
1621	*Klebsiella pneumoniae*	ESBL
1622	*Escherichia coli*	ESBL
1623	*Escherichia coli*	ESBL
1624	*Escherichia coli*	ESBL
1625	*Escherichia coli*	ESBL
1626	*Klebsiella pneumoniae*	NDM
1627	*Escherichia coli*	ESBL
1628	*Klebsiella pneumoniae*	ESBL
1629	*Staphylococcus aureus*	MRSA
1630	*Staphylococcus aureus*	MRSA
1631	*Escherichia coli*	ESBL
1632	*Escherichia coli*	ESBL
1633	*Klebsiella pneumoniae*	ESBL
1634	*Escherichia coli*	ESBL
1635	*Klebsiella pneumoniae*	ESBL
1636	*Escherichia coli*	ESBL
1637	*Escherichia coli*	ESBL
1638	*Klebsiella pneumoniae*	ESBL
1639	*Escherichia coli*	ESBL
1640	*Escherichia coli*	ESBL
1641	*Escherichia coli*	ESBL
1642	*Enterobacter cloacae complex*	VIM
1643	*Staphylococcus aureus*	MRSA
1644	*Escherichia coli*	ESBL
1645	*Staphylococcus aureus*	MRSA
1646	*Escherichia coli*	ESBL
1647	*Staphylococcus aureus*	MRSA
1648	*Acinetobacter baumannii*	OXA23
1649	*Klebsiella pneumoniae*	ESBL
1650	*Escherichia coli*	ESBL
1651	*Escherichia coli*	ESBL
1652	*Escherichia coli*	OXA48
1653	*Citrobacter freundii*	OXA48
1654	*Escherichia coli*	ESBL

1655	*Staphylococcus aureus*	MRSA
1656	*Escherichia coli*	ESBL
1657	*Staphylococcus aureus*	MRSA
1658	*Escherichia coli*	ESBL
1659	*Escherichia coli*	ESBL
1660	*Escherichia coli*	ESBL
1661	*Escherichia coli*	ESBL
1662	*Escherichia coli*	OXA48
1663	*Klebsiella pneumoniae*	ESBL
1664	*Escherichia coli*	ESBL
1665	*Klebsiella pneumoniae*	ESBL
1666	*Escherichia coli*	ESBL
1667	*Klebsiella pneumoniae*	ESBL
1668	*Klebsiella pneumoniae*	ESBL
1669	*Klebsiella pneumoniae*	ESBL
1670	*Escherichia coli*	ESBL
1671	*Staphylococcus aureus*	MRSA
1672	*Escherichia coli*	ESBL
1673	*Escherichia coli*	ESBL
1674	*Escherichia coli*	ESBL
1675	*Escherichia coli*	ESBL
1676	*Enterobacter cloacae complex*	OXA48
1677	*Klebsiella pneumoniae*	ESBL
1678	*Escherichia coli*	OXA48
1679	*Citrobacter freundii*	OXA48
1680	*Escherichia coli*	ESBL
1681	*Klebsiella pneumoniae*	OXA48
1682	*Enterococcus faecium*	VanA
1683	*Acinetobacter baumannii*	OXA23
1684	*Escherichia coli*	ESBL
1685	*Escherichia coli*	OXA48
1686	*Enterobacter cloacae complex*	VIM
1687	*Escherichia coli*	ESBL
1688	*Escherichia coli*	ESBL
1689	*Escherichia coli*	ESBL
1690	*Klebsiella pneumoniae*	ESBL
1691	*Escherichia coli*	ESBL
1692	*Acinetobacter baumannii*	OXA23
1693	*Escherichia coli*	ESBL
1694	*Escherichia coli*	ESBL
1695	*Escherichia coli*	ESBL
1696	*Klebsiella pneumoniae*	ESBL
1697	*Pseudomonas putida*	NDM, VIM
1698	*Klebsiella pneumoniae*	ESBL
1699	*Staphylococcus aureus*	MRSA
1700	*Escherichia coli*	ESBL

1701	*Klebsiella pneumoniae*	ESBL
1702	*Klebsiella pneumoniae*	ESBL
1703	*Klebsiella pneumoniae*	ESBL
1704	*Escherichia coli*	ESBL
1705	*Escherichia coli*	ESBL
1706	*Citrobacter freundii*	ESBL
1707	*Escherichia coli*	OXA48
1708	*Escherichia coli*	ESBL
1709	*Klebsiella pneumoniae*	ESBL
1710	*Staphylococcus aureus*	MRSA
1711	*Klebsiella pneumoniae*	ESBL
1712	*Klebsiella pneumoniae*	ESBL
1713	*Escherichia coli*	ESBL
1714	*Escherichia coli*	ESBL
1715	*Klebsiella pneumoniae*	ESBL
1716	*Staphylococcus aureus*	MRSA
1717	*Staphylococcus aureus*	MRSA
1718	*Klebsiella pneumoniae*	ESBL
1719	*Staphylococcus aureus*	MRSA
1720	*Escherichia coli*	ESBL
1721	*Klebsiella pneumoniae*	ESBL
1722	*Klebsiella aerogenes*	ESBL
1723	*Escherichia coli*	ESBL
1724	*Klebsiella pneumoniae*	ESBL
1725	*Klebsiella pneumoniae*	NDM
1726	*Staphylococcus aureus*	MRSA
1727	*Escherichia coli*	ESBL
1728	*Staphylococcus aureus*	MRSA
1729	*Klebsiella pneumoniae*	ESBL
1730	*Klebsiella pneumoniae*	ESBL
1731	*Klebsiella pneumoniae*	ESBL
1732	*Escherichia coli*	ESBL
1733	*Escherichia coli*	ESBL
1734	*Staphylococcus aureus*	MRSA
1735	*Enterobacter cloacae complex*	ESBL
1736	*Escherichia coli*	ESBL
1737	*Escherichia coli*	ESBL
1738	*Escherichia coli*	ESBL
1739	*Klebsiella aerogenes*	ESBL
1740	*Escherichia coli*	ESBL
1741	*Escherichia coli*	OXA48
1742	*Escherichia coli*	OXA48
1743	*Klebsiella pneumoniae*	OXA48
1744	*Escherichia coli*	ESBL
1745	*Escherichia coli*	ESBL
1746	*Klebsiella pneumoniae*	NDM, OXA48

1747	*Escherichia coli*	NDM
1748	*Staphylococcus aureus*	MRSA
1749	*Enterobacter cloacae complex*	ESBL
1750	*Klebsiella pneumoniae*	ESBL
1751	*Escherichia coli*	ESBL
1752	*Klebsiella pneumoniae*	ESBL
1753	*Klebsiella pneumoniae*	ESBL
1754	*Escherichia coli*	ESBL
1755	*Citrobacter freundii*	ESBL
1756	*Citrobacter freundii*	NDM, OXA48
1757	*Klebsiella pneumoniae*	NDM, OXA48
1758	*Escherichia coli*	ESBL
1759	*Escherichia coli*	ESBL
1760	*Escherichia coli*	ESBL
1761	*Escherichia coli*	ESBL
1762	*Klebsiella pneumoniae*	ESBL
1763	*Staphylococcus aureus*	MRSA
1764	*Escherichia coli*	OXA48
1765	*Klebsiella pneumoniae*	ESBL
1766	*Klebsiella pneumoniae*	ESBL
1767	*Escherichia coli*	ESBL
1768	*Escherichia coli*	ESBL
1769	*Escherichia coli*	ESBL
1770	*Enterobacter cloacae complex*	ESBL
1771	*Escherichia coli*	ESBL
1772	*Escherichia coli*	NDM
1773	*Escherichia coli*	ESBL
1774	*Citrobacter freundii*	OXA48
1775	*Klebsiella pneumoniae*	OXA48
1776	*Escherichia coli*	ESBL
1777	*Klebsiella pneumoniae*	ESBL
1778	*Escherichia coli*	ESBL
1779	*Escherichia coli*	ESBL
1780	*Escherichia coli*	ESBL
1781	*Klebsiella pneumoniae*	ESBL
1782	*Staphylococcus aureus*	MRSA
1783	*Escherichia coli*	ESBL
1784	*Staphylococcus aureus*	MRSA
1785	*Escherichia coli*	ESBL
1786	*Klebsiella pneumoniae*	ESBL
1787	*Escherichia coli*	OXA48
1788	*Klebsiella pneumoniae*	OXA48
1789	*Klebsiella pneumoniae*	ESBL
1790	*Escherichia coli*	ESBL
1791	*Klebsiella oxytoca*	OXA48

1792	*Staphylococcus aureus*	MRSA
1793	*Escherichia coli*	ESBL
1794	*Escherichia coli*	ESBL
1795	*Klebsiella aerogenes*	ESBL
1796	*Escherichia coli*	ESBL
1797	*Staphylococcus aureus*	MRSA
1798	*Escherichia coli*	ESBL
1799	*Klebsiella pneumoniae*	ESBL
1800	*Klebsiella pneumoniae*	ESBL
1801	*Escherichia coli*	ESBL
1802	*Escherichia coli*	ESBL
1803	*Klebsiella pneumoniae*	NDM
1804	*Escherichia coli*	ESBL
1805	*Citrobacter koseri*	OXA48
1806	*Escherichia coli*	ESBL
1807	*Escherichia coli*	ESBL
1808	*Staphylococcus aureus*	MRSA
1809	*Escherichia coli*	ESBL
1810	*Klebsiella pneumoniae*	ESBL
1811	*Escherichia coli*	ESBL
1812	*Citrobacter freundii*	ESBL
1813	*Citrobacter freundii*	ESBL
1814	*Klebsiella pneumoniae*	ESBL
1815	*Escherichia coli*	ESBL
1816	*Escherichia coli*	ESBL
1817	*Klebsiella pneumoniae*	ESBL
1818	*Escherichia coli*	OXA48
1819	*Escherichia coli*	ESBL
1820	*Escherichia coli*	ESBL
1821	*Klebsiella pneumoniae*	ESBL
1822	*Escherichia coli*	ESBL
1823	*Staphylococcus aureus*	MRSA
1824	*Escherichia coli*	OXA48
1825	*Escherichia coli*	ESBL
1826	*Escherichia coli*	ESBL
1827	*Klebsiella pneumoniae*	ESBL
1828	*Citrobacter freundii*	ESBL
1829	*Escherichia coli*	ESBL
1830	*Klebsiella oxytoca*	ESBL
1831	*Escherichia coli*	NDM
1832	*Klebsiella pneumoniae*	NDM
1833	*Klebsiella pneumoniae*	ESBL
1834	*Klebsiella pneumoniae*	ESBL
1835	*Escherichia coli*	ESBL
1836	*Escherichia coli*	ESBL
1837	*Escherichia coli*	ESBL

1838	*Klebsiella pneumoniae*	ESBL
1839	*Escherichia coli*	ESBL
1840	*Klebsiella pneumoniae*	ESBL
1841	*Staphylococcus aureus*	MRSA
1842	*Klebsiella pneumoniae*	ESBL
1843	*Citrobacter freundii*	ESBL
1844	*Escherichia coli*	ESBL
1845	*Enterobacter cloacae complex*	ESBL
1846	*Klebsiella pneumoniae*	ESBL
1847	*Klebsiella pneumoniae*	ESBL
1848	*Klebsiella pneumoniae*	ESBL
1849	*Escherichia coli*	ESBL
1850	*Escherichia coli*	ESBL
1851	*Klebsiella pneumoniae*	ESBL
1852	*Klebsiella pneumoniae*	ESBL
1853	*Citrobacter freundii*	OXA48
1854	*Klebsiella pneumoniae*	OXA48
1855	*Escherichia coli*	ESBL
1856	*Escherichia coli*	OXA48
1857	*Klebsiella pneumoniae*	OXA48
1858	*Escherichia coli*	OXA48
1859	*Escherichia coli*	ESBL
1860	*Klebsiella pneumoniae*	ESBL
1861	*Acinetobacter baumannii*	OXA23
1862	*Enterobacter cloacae complex*	NDM
1863	*Escherichia coli*	NDM
1864	*Acinetobacter baumannii*	OXA23
1865	*Klebsiella pneumoniae*	ESBL
1866	*Klebsiella pneumoniae*	ESBL
1867	*Escherichia coli*	ESBL
1868	*Escherichia coli*	ESBL
1869	*Citrobacter freundii*	OXA48
1870	*Enterobacter cloacae complex*	ESBL
1871	*Escherichia coli*	ESBL
1872	*Escherichia coli*	OXA48
1873	*Acinetobacter baumannii*	OXA23
1874	*Staphylococcus aureus*	MRSA
1875	*Staphylococcus aureus*	MRSA
1876	*Escherichia coli*	ESBL
1877	*Escherichia coli*	ESBL
1878	*Escherichia coli*	ESBL
1879	*Citrobacter freundii*	OXA48
1880	*Klebsiella pneumoniae*	OXA48
1881	*Escherichia coli*	OXA48
1882	*Escherichia coli*	ESBL
1883	*Escherichia coli*	ESBL

1884	*Staphylococcus aureus*	MRSA
1885	*Klebsiella pneumoniae*	ESBL
1886	*Citrobacter freundii*	ESBL
1887	*Escherichia coli*	ESBL
1888	*Klebsiella pneumoniae*	ESBL
1889	*Klebsiella pneumoniae*	NDM, OXA48
1890	*Klebsiella pneumoniae*	ESBL
1891	*Proteus sp*	ESBL
1892	*Escherichia coli*	ESBL
1893	*Enterobacter cloacae complex*	OXA48
1894	*Klebsiella pneumoniae*	ESBL
1895	*Escherichia coli*	ESBL
1896	*Klebsiella pneumoniae*	ESBL
1897	*Klebsiella pneumoniae*	ESBL
1898	*Escherichia coli*	ESBL
1899	*Klebsiella pneumoniae*	ESBL
1900	*Citrobacter freundii*	OXA48
1901	*Escherichia coli*	ESBL
1902	*Escherichia coli*	ESBL
1903	*Escherichia coli*	ESBL
1904	*Klebsiella pneumoniae*	ESBL
1905	*Escherichia coli*	ESBL
1906	*Escherichia coli*	ESBL
1907	*Staphylococcus aureus*	MRSA
1908	*Escherichia coli*	ESBL
1909	*Klebsiella pneumoniae*	ESBL
1910	*Klebsiella pneumoniae*	OXA48
1911	*Escherichia coli*	OXA48
1912	*Klebsiella pneumoniae*	OXA48
1913	*Klebsiella pneumoniae*	ESBL
1914	*Escherichia coli*	ESBL
1915	*Escherichia coli*	ESBL
1916	*Klebsiella pneumoniae*	ESBL
1917	*Klebsiella pneumoniae*	ESBL
1918	*Staphylococcus aureus*	MRSA
1919	*Klebsiella pneumoniae*	ESBL
1920	*Klebsiella pneumoniae*	ESBL
1921	*Escherichia coli*	ESBL
1922	*Escherichia coli*	ESBL
1923	*Escherichia coli*	ESBL
1924	*Escherichia coli*	ESBL
1925	*Staphylococcus aureus*	MRSA
1926	*Klebsiella pneumoniae*	ESBL
1927	*Escherichia coli*	ESBL
1928	*Klebsiella pneumoniae*	ESBL
1929	*Acinetobacter baumannii*	OXA23

1930	*Klebsiella pneumoniae*	ESBL
1931	*Citrobacter freundii*	ESBL
1932	*Escherichia coli*	ESBL
1933	*Enterobacter cloacae complex*	ESBL
1934	*Enterobacter cloacae complex*	ESBL
1935	*Escherichia coli*	ESBL
1936	*Klebsiella pneumoniae*	ESBL
1937	*Klebsiella pneumoniae*	NDM
1938	*Klebsiella pneumoniae*	ESBL
1939	*Escherichia coli*	ESBL
1940	*Staphylococcus aureus*	MRSA
1941	*Acinetobacter baumannii*	OXA23
1942	*Escherichia coli*	ESBL
1943	*Klebsiella pneumoniae*	ESBL
1944	*Klebsiella pneumoniae*	ESBL
1945	*Enterobacter cloacae complex*	VIM
1946	*Citrobacter freundii*	ESBL
1947	*Enterobacter cloacae complex*	ESBL
1948	*Enterobacter cloacae complex*	ESBL
1949	*Staphylococcus aureus*	MRSA
1950	*Escherichia coli*	ESBL
1951	*Citrobacter freundii*	ESBL
1952	*Staphylococcus aureus*	MRSA
1953	*Klebsiella pneumoniae*	ESBL
1954	*Escherichia coli*	ESBL
1955	*Klebsiella pneumoniae*	ESBL
1956	*Enterobacter cloacae complex*	ESBL
1957	*Escherichia coli*	ESBL
1958	*Klebsiella pneumoniae*	ESBL
1959	*Klebsiella pneumoniae*	ESBL
1960	*Escherichia coli*	ESBL
1961	*Escherichia coli*	ESBL
1962	*Klebsiella pneumoniae*	ESBL
1963	*Escherichia coli*	ESBL
1964	*Klebsiella pneumoniae*	ESBL
1965	*Escherichia coli*	ESBL
1966	*Pseudomonas aeruginosa*	VIM
1967	*Escherichia coli*	ESBL
1968	*Klebsiella pneumoniae*	ESBL
1969	*Enterobacter cloacae complex*	ESBL
1970	*Escherichia coli*	ESBL
1971	*Acinetobacter baumannii*	OXA23
1972	*Escherichia coli*	ESBL
1973	*Klebsiella pneumoniae*	OXA48
1974	*Klebsiella pneumoniae*	ESBL
1975	*Klebsiella pneumoniae*	ESBL

1976	*Enterobacter cloacae complex*	ESBL
1977	*Klebsiella pneumoniae*	ESBL
1978	*Escherichia coli*	ESBL
1979	*Staphylococcus aureus*	MRSA
1980	*Klebsiella pneumoniae*	ESBL
1981	*Escherichia coli*	ESBL
1982	*Escherichia coli*	OXA48
1983	*Klebsiella pneumoniae*	OXA48
1984	*Escherichia coli*	OXA48
1985	*Escherichia coli*	ESBL
1986	*Staphylococcus aureus*	MRSA
1987	*Klebsiella pneumoniae*	OXA48
1988	*Escherichia coli*	ESBL
1989	*Klebsiella pneumoniae*	NDM
1990	*Klebsiella pneumoniae*	ESBL
1991	*Escherichia coli*	ESBL
1992	*Escherichia coli*	ESBL
1993	*Escherichia coli*	ESBL
1994	*Escherichia coli*	ESBL
1995	*Pseudomonas aeruginosa*	ESBL
1996	*Klebsiella pneumoniae*	NDM
1997	*Klebsiella pneumoniae*	ESBL
1998	*Enterobacter cloacae complex*	ESBL
1999	*Klebsiella pneumoniae*	ESBL
2000	*Escherichia coli*	ESBL
2001	*Escherichia coli*	ESBL
2002	*Acinetobacter baumannii*	OXA23
2003	*Klebsiella oxytoca*	ESBL
2004	*Escherichia coli*	ESBL
2005	*Staphylococcus aureus*	MRSA
2006	*Citrobacter freundii*	ESBL
2007	*Klebsiella pneumoniae*	ESBL
2008	*Escherichia coli*	OXA48
2009	*Klebsiella oxytoca*	OXA48
2010	*Klebsiella oxytoca*	ESBL
2011	*Escherichia coli*	ESBL
2012	*Escherichia coli*	ESBL
2013	*Escherichia coli*	ESBL
2014	*Acinetobacter baumannii*	OXA23
2015	*Klebsiella pneumoniae*	ESBL
2016	*Klebsiella pneumoniae*	ESBL
2017	*Escherichia coli*	ESBL
2018	*Escherichia coli*	ESBL
2019	*Klebsiella pneumoniae*	ESBL
2020	*Enterobacter cloacae complex*	VIM
2021	*Escherichia coli*	ESBL

2022	*Escherichia coli*	ESBL
2023	*Staphylococcus aureus*	MRSA
2024	*Serratia marcescens*	ESBL
2025	*Klebsiella pneumoniae*	ESBL
2026	*Klebsiella pneumoniae*	ESBL
2027	*Klebsiella pneumoniae*	ESBL
2028	*Klebsiella pneumoniae*	ESBL
2029	*Klebsiella pneumoniae*	ESBL
2030	*Escherichia coli*	ESBL
2031	*Klebsiella pneumoniae*	ESBL
2032	*Escherichia coli*	ESBL
2033	*Klebsiella pneumoniae*	ESBL
2034	*Escherichia coli*	ESBL
2035	*Klebsiella pneumoniae*	ESBL
2036	*Citrobacter freundii*	OXA48
2037	*Staphylococcus aureus*	MRSA
2038	*Escherichia coli*	ESBL
2039	*Klebsiella pneumoniae*	ESBL
2040	*Escherichia coli*	ESBL
2041	*Klebsiella pneumoniae*	ESBL
2042	*Enterobacter cloacae complex*	ESBL
2043	*Escherichia coli*	ESBL
2044	*Escherichia coli*	ESBL
2045	*Citrobacter freundii*	ESBL
2046	*Staphylococcus aureus*	MRSA
2047	*Acinetobacter baumannii*	OXA23
2048	*Citrobacter braakii*	ESBL
2049	*Enterobacter cloacae complex*	ESBL
2050	*Escherichia coli*	ESBL
2051	*Escherichia coli*	ESBL
2052	*Escherichia coli*	ESBL
2053	*Klebsiella pneumoniae*	ESBL
2054	*Escherichia coli*	ESBL
2055	*Escherichia coli*	ESBL
2056	*Klebsiella pneumoniae*	ESBL
2057	*Klebsiella pneumoniae*	ESBL
2058	*Escherichia coli*	ESBL
2059	*Escherichia coli*	ESBL
2060	*Escherichia coli*	ESBL
2061	*Staphylococcus aureus*	MRSA
2062	*Escherichia coli*	ESBL
2063	*Escherichia coli*	ESBL
2064	*Escherichia coli*	ESBL
2065	*Klebsiella pneumoniae*	OXA48
2066	*Klebsiella pneumoniae*	ESBL
2067	*Escherichia coli*	ESBL

2068	*Escherichia coli*	ESBL
2069	*Escherichia coli*	ESBL
2070	*Staphylococcus aureus*	MRSA
2071	*Klebsiella pneumoniae*	ESBL
2072	*Escherichia coli*	ESBL
2073	*Klebsiella pneumoniae*	ESBL
2074	*Escherichia coli*	ESBL
2075	*Escherichia coli*	ESBL
2076	*Klebsiella pneumoniae*	ESBL
2077	*Escherichia coli*	ESBL
2078	*Klebsiella pneumoniae*	ESBL
2079	*Klebsiella pneumoniae*	ESBL
2080	*Escherichia coli*	ESBL
2081	*Escherichia coli*	ESBL
2082	*Klebsiella pneumoniae*	ESBL
2083	*Escherichia coli*	ESBL
2084	*Klebsiella pneumoniae*	ESBL
2085	*Escherichia coli*	ESBL
2086	*Klebsiella pneumoniae*	NDM, OXA48
2087	*Klebsiella oxytoca*	ESBL
2088	*Klebsiella pneumoniae*	ESBL
2089	*Escherichia coli*	ESBL
2090	*Escherichia coli*	ESBL
2091	*Klebsiella oxytoca*	OXA48
2092	*Enterobacter cloacae complex*	ESBL
2093	*Escherichia coli*	ESBL
2094	*Escherichia coli*	ESBL
2095	*Klebsiella pneumoniae*	ESBL
2096	*Escherichia coli*	ESBL
2097	*Escherichia coli*	ESBL
2098	*Staphylococcus aureus*	MRSA
2099	*Escherichia coli*	ESBL
2100	*Escherichia coli*	ESBL
2101	*Klebsiella oxytoca*	ESBL
2102	*Klebsiella pneumoniae*	ESBL
2103	*Klebsiella pneumoniae*	ESBL
2104	*Escherichia coli*	ESBL
2105	*Klebsiella pneumoniae*	ESBL
2106	*Escherichia coli*	ESBL
2107	*Klebsiella pneumoniae*	ESBL
2108	*Escherichia coli*	ESBL
2109	*Enterobacter cloacae complex*	ESBL
2110	*Escherichia coli*	ESBL
2111	*Escherichia coli*	ESBL
2112	*Staphylococcus aureus*	MRSA
2113	*Klebsiella pneumoniae*	NDM

2114	*Escherichia coli*	ESBL
2115	*Escherichia coli*	ESBL
2116	*Escherichia coli*	ESBL
2117	*Klebsiella pneumoniae*	ESBL
2118	*Escherichia coli*	ESBL
2119	*Escherichia coli*	ESBL
2120	*Escherichia coli*	ESBL
2121	*Staphylococcus aureus*	MRSA
2122	*Escherichia coli*	ESBL
2123	*Klebsiella pneumoniae*	ESBL
2124	*Escherichia coli*	ESBL
2125	*Klebsiella pneumoniae*	NDM, OXA48
2126	*Klebsiella pneumoniae*	ESBL
2127	*Enterococcus faecium*	VanA
2128	*Enterobacter cloacae complex*	ESBL
2129	*Klebsiella pneumoniae*	ESBL
2130	*Citrobacter freundii*	ESBL
2131	*Escherichia coli*	ESBL
2132	*Klebsiella pneumoniae*	ESBL
2133	*Klebsiella variicola*	ESBL
2134	*Staphylococcus aureus*	MRSA
2135	*Klebsiella pneumoniae*	ESBL
2136	*Enterobacter cloacae complex*	ESBL
2137	*Enterobacter cloacae complex*	ESBL
2138	*Enterobacter cloacae complex*	ESBL
2139	*Enterobacter cloacae complex*	ESBL
2140	*Enterobacter cloacae complex*	NDM, OXA48
2141	*Klebsiella pneumoniae*	NDM, OXA48
2142	*Escherichia coli*	ESBL
2143	*Escherichia coli*	ESBL
2144	*Escherichia coli*	ESBL
2145	*Escherichia coli*	ESBL
2146	*Staphylococcus aureus*	MRSA
2147	*Escherichia coli*	ESBL
2148	*Klebsiella pneumoniae*	ESBL
2149	*Escherichia coli*	ESBL
2150	*Klebsiella pneumoniae*	ESBL
2151	*Staphylococcus aureus*	MRSA
2152	*Klebsiella pneumoniae*	ESBL
2153	*Acinetobacter baumannii*	OXA23
2154	*Staphylococcus aureus*	MRSA
2155	*Klebsiella pneumoniae*	ESBL
2156	*Klebsiella pneumoniae*	ESBL
2157	*Klebsiella pneumoniae*	NDM, OXA48

2158	*Enterococcus faecium*	VanA
2159	*Escherichia coli*	NDM, OXA48
2160	*Klebsiella pneumoniae*	ESBL
2161	*Enterobacter cloacae complex*	ESBL
2162	*Escherichia coli*	ESBL
2163	*Enterobacter cloacae complex*	ESBL
2164	*Escherichia coli*	ESBL
2165	*Escherichia coli*	ESBL
2166	*Escherichia coli*	ESBL
2167	*Citrobacter freundii*	ESBL
2168	*Klebsiella pneumoniae*	ESBL
2169	*Klebsiella pneumoniae*	ESBL
2170	*Escherichia coli*	ESBL
2171	*Klebsiella pneumoniae*	ESBL
2172	*Escherichia coli*	ESBL
2173	*Klebsiella pneumoniae*	ESBL
2174	*Staphylococcus aureus*	MRSA
2175	*Klebsiella oxytoca*	ESBL
2176	*Enterobacter cloacae complex*	VIM
2177	*Klebsiella pneumoniae*	ESBL
2178	*Escherichia coli*	ESBL
2179	*Klebsiella pneumoniae*	NDM, OXA48
2180	*Escherichia coli*	NDM
2181	*Enterobacter cloacae complex*	ESBL
2182	*Escherichia coli*	ESBL
2183	*Escherichia coli*	OXA48
2184	*Escherichia coli*	ESBL
2185	*Escherichia coli*	ESBL
2186	*Escherichia coli*	ESBL
2187	*Escherichia coli*	ESBL
2188	*Escherichia coli*	ESBL
2189	*Enterobacter cloacae complex*	ESBL
2190	*Acinetobacter baumannii*	OXA23
2191	*Escherichia coli*	ESBL
2192	*Klebsiella pneumoniae*	OXA48
2193	*Klebsiella pneumoniae*	ESBL
2194	*Staphylococcus aureus*	MRSA
2195	*Enterococcus faecium*	VanA
2196	*Enterobacter cloacae complex*	ESBL
2197	*Citrobacter freundii*	VIM
2198	*Klebsiella pneumoniae*	OXA48
2199	*Escherichia coli*	OXA48
2200	*Klebsiella pneumoniae*	ESBL
2201	*Staphylococcus aureus*	MRSA
2202	*Staphylococcus aureus*	MRSA

2203	*Klebsiella pneumoniae*	ESBL
2204	*Acinetobacter baumannii*	OXA23
2205	*Enterobacter cloacae complex*	ESBL
2206	*Escherichia coli*	ESBL
2207	*Escherichia coli*	OXA48
2208	*Klebsiella pneumoniae*	OXA48
2209	*Klebsiella pneumoniae*	ESBL
2210	*Klebsiella pneumoniae*	ESBL
2211	*Citrobacter freundii*	ESBL
2212	*Enterobacter cloacae complex*	ESBL
2213	*Klebsiella pneumoniae*	ESBL
2214	*Klebsiella pneumoniae*	ESBL
2215	*Escherichia coli*	ESBL
2216	*Escherichia coli*	NDM
2217	*Klebsiella pneumoniae*	ESBL
2218	*Escherichia coli*	ESBL
2219	*Escherichia coli*	ESBL
2220	*Enterobacter cloacae complex*	ESBL
2221	*Staphylococcus aureus*	MRSA
2222	*Enterobacter cloacae complex*	VIM
2223	*Escherichia coli*	ESBL
2224	*Klebsiella pneumoniae*	NDM, VIM
2225	*Enterobacter cloacae complex*	NDM, VIM
2226	*Escherichia coli*	ESBL
2227	*Citrobacter freundii*	ESBL
2228	*Klebsiella pneumoniae*	ESBL
2229	*Escherichia coli*	ESBL
2230	*Staphylococcus aureus*	MRSA
2231	*Escherichia coli*	ESBL
2232	*Citrobacter amalonaticus*	ESBL
2233	*Escherichia coli*	ESBL
2234	*Citrobacter freundii*	ESBL
2235	*Escherichia coli*	ESBL
2236	*Staphylococcus aureus*	MRSA
2237	*Escherichia coli*	ESBL
2238	*Escherichia coli*	ESBL
2239	*Escherichia coli*	ESBL
2240	*Escherichia coli*	ESBL
2241	*Klebsiella pneumoniae*	ESBL
2242	*Escherichia coli*	ESBL
2243	*Escherichia coli*	ESBL
2244	*Escherichia coli*	ESBL
2245	*Staphylococcus aureus*	MRSA
2246	*Staphylococcus aureus*	MRSA
2247	*Escherichia coli*	ESBL
2248	*Acinetobacter baumannii*	OXA23

2249	*Staphylococcus aureus*	MRSA
2250	*Escherichia coli*	ESBL
2251	*Klebsiella pneumoniae*	ESBL
2252	*Klebsiella pneumoniae*	ESBL
2253	*Klebsiella pneumoniae*	ESBL
2254	*Klebsiella pneumoniae*	ESBL
2255	*Escherichia coli*	ESBL
2256	*Klebsiella pneumoniae*	ESBL
2257	*Staphylococcus aureus*	MRSA
2258	*Escherichia coli*	ESBL
2259	*Klebsiella pneumoniae*	ESBL
2260	*Escherichia coli*	ESBL
2261	*Escherichia coli*	ESBL
2262	*Escherichia coli*	ESBL
2263	*Klebsiella pneumoniae*	ESBL
2264	*Escherichia coli*	ESBL
2265	*Klebsiella pneumoniae*	ESBL
2266	*Escherichia coli*	ESBL
2267	*Escherichia coli*	ESBL
2268	*Escherichia coli*	NDM
2269	*Klebsiella pneumoniae*	NDM
2270	*Escherichia coli*	ESBL
2271	*Staphylococcus aureus*	MRSA
2272	*Staphylococcus aureus*	MRSA
2273	*Escherichia coli*	ESBL
2274	*Escherichia coli*	ESBL
2275	*Escherichia coli*	ESBL
2276	*Klebsiella pneumoniae*	ESBL
2277	*Escherichia coli*	ESBL
2278	*Escherichia coli*	ESBL
2279	*Klebsiella pneumoniae*	ESBL
2280	*Staphylococcus aureus*	MRSA
2281	*Escherichia coli*	ESBL
2282	*Escherichia coli*	ESBL
2283	*Klebsiella pneumoniae*	ESBL
2284	*Escherichia coli*	ESBL
2285	*Escherichia coli*	ESBL
2286	*Escherichia coli*	ESBL
2287	*Escherichia coli*	ESBL
2288	*Klebsiella pneumoniae*	ESBL
2289	*Escherichia coli*	ESBL
2290	*Escherichia coli*	ESBL
2291	*Klebsiella pneumoniae*	ESBL
2292	*Citrobacter freundii*	OXA48
2293	*Klebsiella pneumoniae*	ESBL
2294	*Citrobacter koseri*	OXA48

2295	*Escherichia coli*	ESBL
2296	*Enterobacter cloacae complex*	ESBL
2297	*Acinetobacter baumannii*	OXA23
2298	*Citrobacter farmeri*	OXA48
2299	*Escherichia coli*	OXA48
2300	*Staphylococcus aureus*	MRSA
2301	*Escherichia coli*	ESBL
2302	*Citrobacter freundii*	ESBL
2303	*Escherichia coli*	OXA48
2304	*Klebsiella pneumoniae*	NDM, OXA48
2305	*Escherichia coli*	ESBL
2306	*Klebsiella pneumoniae*	ESBL
2307	*Enterobacter cloacae complex*	ESBL
2308	*Klebsiella pneumoniae*	ESBL
2309	*Escherichia coli*	ESBL
2310	*Klebsiella pneumoniae*	ESBL
2311	*Staphylococcus aureus*	MRSA
2312	*Klebsiella pneumoniae*	ESBL
2313	*Klebsiella pneumoniae*	ESBL
2314	*Escherichia coli*	ESBL
2315	*Klebsiella pneumoniae*	ESBL
2316	*Enterobacter cloacae complex*	ESBL
2317	*Klebsiella pneumoniae*	ESBL
2318	*Escherichia coli*	ESBL
2319	*Klebsiella pneumoniae*	ESBL
2320	*Enterobacter cloacae complex*	ESBL
2321	*Klebsiella pneumoniae*	ESBL
2322	*Enterobacter cloacae complex*	ESBL
2323	*Klebsiella pneumoniae*	ESBL
2324	*Enterobacter cloacae complex*	ESBL
2325	*Escherichia coli*	ESBL
2326	*Citrobacter freundii*	ESBL
2327	*Klebsiella pneumoniae*	ESBL
2328	*Escherichia coli*	ESBL
2329	*Citrobacter freundii*	ESBL
2330	*Klebsiella pneumoniae*	ESBL
2331	*Escherichia coli*	ESBL
2332	*Escherichia coli*	ESBL
2333	*Klebsiella pneumoniae*	ESBL
2334	*Escherichia coli*	ESBL
2335	*Klebsiella pneumoniae*	ESBL
2336	*Enterobacter cloacae complex*	ESBL
2337	*Escherichia coli*	ESBL
2338	*Klebsiella pneumoniae*	ESBL
2339	*Klebsiella pneumoniae*	ESBL
2340	*Klebsiella pneumoniae*	ESBL

2341	*Klebsiella pneumoniae*	ESBL
2342	*Klebsiella pneumoniae*	ESBL
2343	*Enterococcus faecium*	VanB
2344	*Klebsiella pneumoniae*	NDM, OXA48
2345	*Escherichia coli*	ESBL
2346	*Escherichia coli*	ESBL
2347	*Klebsiella pneumoniae*	ESBL
2348	*Enterobacter cloacae complex*	ESBL
2349	*Klebsiella pneumoniae*	ESBL
2350	*Citrobacter koseri*	ESBL
2351	*Citrobacter braakii*	ESBL
2352	*Escherichia coli*	ESBL
2353	*Klebsiella pneumoniae*	ESBL
2354	*Escherichia coli*	ESBL
2355	*Acinetobacter baumannii*	OXA23
2356	*Staphylococcus aureus*	MRSA
2357	*Klebsiella pneumoniae*	ESBL
2358	*Escherichia coli*	ESBL
2359	*Escherichia coli*	ESBL
2360	*Escherichia coli*	ESBL
2361	*Enterobacter cloacae complex*	ESBL
2362	*Escherichia coli*	ESBL
2363	*Klebsiella pneumoniae*	ESBL
2364	*Staphylococcus aureus*	MRSA
2365	*Escherichia coli*	ESBL
2366	*Klebsiella pneumoniae*	ESBL
2367	*Klebsiella pneumoniae*	ESBL
2368	*Klebsiella pneumoniae*	NDM
2369	*Staphylococcus aureus*	MRSA
2370	*Klebsiella pneumoniae*	ESBL
2371	*Klebsiella pneumoniae*	ESBL
2372	*Escherichia coli*	ESBL
2373	*Escherichia coli*	ESBL
2374	*Enterobacter cloacae complex*	ESBL
2375	*Acinetobacter baumannii*	OXA23
2376	*Acinetobacter baumannii*	OXA23
2377	*Klebsiella pneumoniae*	ESBL
2378	*Klebsiella pneumoniae*	ESBL
2379	*Klebsiella pneumoniae*	ESBL
2380	*Staphylococcus aureus*	MRSA
2381	*Escherichia coli*	ESBL
2382	*Escherichia coli*	ESBL
2383	*Escherichia coli*	ESBL
2384	*Escherichia coli*	ESBL
2385	*Klebsiella pneumoniae*	VIM
2386	*Klebsiella pneumoniae*	ESBL

2387	*Klebsiella pneumoniae*	OXA48
2388	*Acinetobacter baumannii*	OXA23
2389	*Klebsiella pneumoniae*	NDM
2390	*Klebsiella pneumoniae*	ESBL
2391	*Acinetobacter baumannii*	OXA23
2392	*Escherichia coli*	ESBL
2393	*Klebsiella pneumoniae*	ESBL
2394	*Escherichia coli*	ESBL
2395	*Klebsiella pneumoniae*	ESBL
2396	*Klebsiella pneumoniae*	ESBL
2397	*Klebsiella pneumoniae*	ESBL
2398	*Staphylococcus aureus*	MRSA
2399	*Klebsiella pneumoniae*	ESBL
2400	*Escherichia coli*	ESBL
2401	*Klebsiella pneumoniae*	ESBL
2402	*Escherichia coli*	ESBL
2403	*Escherichia coli*	ESBL
2404	*Escherichia coli*	ESBL
2405	*Escherichia coli*	ESBL
2406	*Escherichia coli*	ESBL
2407	*Escherichia coli*	ESBL
2408	*Escherichia coli*	ESBL
2409	*Klebsiella pneumoniae*	NDM
2410	*Escherichia coli*	ESBL
2411	*Escherichia coli*	ESBL
2412	*Escherichia coli*	ESBL
2413	*Klebsiella pneumoniae*	NDM
2414	*Escherichia coli*	ESBL
2415	*Escherichia coli*	ESBL
2416	*Staphylococcus aureus*	MRSA
2417	*Escherichia coli*	ESBL
2418	*Enterobacter cloacae complex*	VIM
2419	*Escherichia coli*	ESBL
2420	*Escherichia coli*	ESBL
2421	*Escherichia coli*	ESBL
2422	*Escherichia coli*	ESBL
2423	*Enterobacter cloacae complex*	ESBL
2424	*Citrobacter freundii*	ESBL
2425	*Klebsiella pneumoniae*	ESBL
2426	*Klebsiella pneumoniae*	ESBL
2427	*Klebsiella pneumoniae*	ESBL
2428	*Klebsiella pneumoniae*	ESBL
2429	*Escherichia coli*	ESBL
2430	*Escherichia coli*	ESBL
2431	*Klebsiella pneumoniae*	ESBL
2432	*Enterobacter cloacae complex*	VIM

2433	*Klebsiella pneumoniae*	ESBL
2434	*Enterobacter cloacae complex*	ESBL
2435	*Escherichia coli*	ESBL
2436	*Klebsiella pneumoniae*	ESBL
2437	*Escherichia coli*	ESBL
2438	*Escherichia coli*	ESBL
2439	*Klebsiella pneumoniae*	ESBL
2440	*Klebsiella pneumoniae*	ESBL
2441	*Escherichia coli*	ESBL
2442	*Staphylococcus aureus*	MRSA
2443	*Morganella morganii*	ESBL
2444	*Escherichia coli*	ESBL
2445	*Acinetobacter baumannii*	OXA23
2446	*Escherichia coli*	ESBL
2447	*Staphylococcus aureus*	MRSA
2448	*Klebsiella pneumoniae*	ESBL
2449	*Escherichia coli*	NDM
2450	*Citrobacter freundii*	OXA48
2451	*Escherichia coli*	OXA48
2452	*Escherichia coli*	ESBL
2453	*Klebsiella pneumoniae*	ESBL
2454	*Citrobacter freundii*	ESBL
2455	*Klebsiella pneumoniae*	ESBL
2456	*Citrobacter freundii*	ESBL
2457	*Escherichia coli*	ESBL
2458	*Escherichia coli*	ESBL
2459	*Escherichia coli*	ESBL
2460	*Escherichia coli*	ESBL
2461	*Escherichia coli*	ESBL
2462	*Enterobacter cloacae complex*	ESBL
2463	*Enterobacter cloacae complex*	ESBL
2464	*Klebsiella pneumoniae*	ESBL
2465	*Escherichia coli*	ESBL
2466	*Escherichia coli*	ESBL
2467	*Escherichia coli*	ESBL
2468	*Escherichia coli*	ESBL
2469	*Escherichia coli*	ESBL
2470	*Escherichia coli*	ESBL
2471	*Klebsiella pneumoniae*	ESBL
2472	*Enterobacter cloacae complex*	ESBL
2473	*Escherichia coli*	ESBL
2474	*Klebsiella pneumoniae*	ESBL
2475	*Citrobacter sedlakii*	ESBL
2476	*Escherichia coli*	ESBL
2477	*Citrobacter freundii*	OXA48
2478	*Escherichia coli*	ESBL

2479	*Escherichia coli*	ESBL
2480	*Klebsiella pneumoniae*	ESBL
2481	*Enterobacter cloacae complex*	VIM
2482	*Staphylococcus aureus*	MRSA
2483	*Escherichia coli*	ESBL
2484	*Klebsiella aerogenes*	ESBL
2485	*Escherichia coli*	ESBL
2486	*Escherichia coli*	ESBL
2487	*Escherichia coli*	ESBL
2488	*Escherichia coli*	ESBL
2489	*Escherichia coli*	ESBL
2490	*Escherichia coli*	ESBL
2491	*Klebsiella pneumoniae*	ESBL
2492	*Escherichia coli*	NDM
2493	*Escherichia coli*	ESBL
2494	*Klebsiella pneumoniae*	ESBL
2495	*Enterobacter cloacae complex*	VIM
2496	*Citrobacter freundii*	ESBL
2497	*Klebsiella pneumoniae*	ESBL
2498	*Escherichia coli*	ESBL
2499	*Klebsiella pneumoniae*	ESBL
2500	*Klebsiella pneumoniae*	ESBL
2501	*Escherichia coli*	ESBL
2502	*Klebsiella pneumoniae*	ESBL
2503	*Staphylococcus aureus*	MRSA
2504	*Escherichia coli*	ESBL
2505	*Escherichia coli*	ESBL
2506	*Escherichia coli*	ESBL
2507	*Escherichia coli*	ESBL
2508	*Klebsiella pneumoniae*	ESBL
2509	*Klebsiella pneumoniae*	ESBL
2510	*Escherichia coli*	NDM
2511	*Klebsiella pneumoniae*	NDM
2512	*Escherichia coli*	NDM, OXA48
2513	*Staphylococcus aureus*	MRSA
2514	*Klebsiella pneumoniae*	ESBL
2515	*Escherichia coli*	ESBL
2516	*Klebsiella pneumoniae*	ESBL
2517	*Escherichia coli*	ESBL
2518	*Escherichia coli*	ESBL
2519	*Escherichia coli*	ESBL
2520	*Escherichia coli*	ESBL
2521	*Escherichia coli*	ESBL
2522	*Escherichia coli*	ESBL
2523	*Enterobacter cloacae complex*	ESBL
2524	*Escherichia coli*	ESBL

2525	*Staphylococcus aureus*	MRSA
2526	*Escherichia coli*	ESBL
2527	*Staphylococcus aureus*	MRSA
2528	*Escherichia coli*	ESBL
2529	*Escherichia coli*	ESBL
2530	*Staphylococcus aureus*	MRSA
2531	*Escherichia coli*	ESBL
2532	*Klebsiella pneumoniae*	ESBL
2533	*Escherichia coli*	ESBL
2534	*Escherichia coli*	ESBL
2535	*Escherichia coli*	ESBL
2536	*Enterobacter cloacae complex*	ESBL
2537	*Escherichia coli*	OXA48
2538	*Klebsiella pneumoniae*	OXA48
2539	*Escherichia coli*	ESBL
2540	*Klebsiella pneumoniae*	ESBL
2541	*Klebsiella pneumoniae*	OXA48
2542	*Enterobacter cloacae complex*	OXA48
2543	*Escherichia coli*	ESBL
2544	*Klebsiella pneumoniae*	ESBL
2545	*Klebsiella pneumoniae*	ESBL
2546	*Escherichia coli*	ESBL
2547	*Escherichia coli*	ESBL
2548	*Escherichia coli*	ESBL
2549	*Escherichia coli*	ESBL
2550	*Escherichia coli*	ESBL
2551	*Klebsiella pneumoniae*	ESBL
2552	*Escherichia coli*	ESBL
2553	*Klebsiella pneumoniae*	ESBL
2554	*Staphylococcus aureus*	MRSA
2555	*Escherichia coli*	ESBL
2556	*Klebsiella pneumoniae*	ESBL
2557	*Escherichia coli*	ESBL
2558	*Escherichia coli*	ESBL
2559	*Staphylococcus aureus*	MRSA
2560	*Escherichia coli*	ESBL
2561	*Klebsiella pneumoniae*	KPC
2562	*Klebsiella pneumoniae*	ESBL
2563	*Klebsiella pneumoniae*	ESBL
2564	*Enterobacter cloacae complex*	ESBL
2565	*Klebsiella pneumoniae*	NDM, OXA48
2566	*Klebsiella pneumoniae*	ESBL
2567	*Klebsiella pneumoniae*	ESBL
2568	*Klebsiella oxytoca*	OXA48
2569	*Escherichia coli*	ESBL
2570	*Escherichia coli*	ESBL

2571	*Staphylococcus aureus*	MRSA
2572	*Citrobacter koseri*	ESBL
2573	*Escherichia coli*	ESBL
2574	*Staphylococcus aureus*	MRSA
2575	*Escherichia coli*	ESBL
2576	*Staphylococcus aureus*	MRSA
2577	*Klebsiella pneumoniae*	OXA48
2578	*Klebsiella pneumoniae*	ESBL
2579	*Escherichia coli*	ESBL
2580	*Escherichia coli*	ESBL
2581	*Staphylococcus aureus*	MRSA
2582	*Klebsiella variicola*	OXA48
2583	*Staphylococcus aureus*	MRSA
2584	*Acinetobacter baumannii*	OXA23
2585	*Klebsiella pneumoniae*	ESBL
2586	*Staphylococcus aureus*	MRSA
2587	*Escherichia coli*	ESBL
2588	*Escherichia coli*	ESBL
2589	*Escherichia coli*	ESBL
2590	*Escherichia coli*	ESBL
2591	*Enterobacter cloacae complex*	ESBL
2592	*Klebsiella pneumoniae*	ESBL
2593	*Acinetobacter baumannii*	OXA23
2594	*Escherichia coli*	ESBL
2595	*Klebsiella pneumoniae*	ESBL
2596	*Klebsiella pneumoniae*	NDM
2597	*Escherichia coli*	ESBL
2598	*Staphylococcus aureus*	MRSA
2599	*Escherichia coli*	ESBL
2600	*Escherichia coli*	ESBL
2601	*Escherichia coli*	ESBL
2602	*Staphylococcus aureus*	MRSA
2603	*Klebsiella pneumoniae*	ESBL
2604	*Escherichia coli*	ESBL
2605	*Klebsiella pneumoniae*	ESBL
2606	*Klebsiella pneumoniae*	ESBL
2607	*Staphylococcus aureus*	MRSA
2608	*Klebsiella pneumoniae*	OXA48
2609	*Klebsiella pneumoniae*	ESBL
2610	*Enterobacter cloacae complex*	ESBL
2611	*Klebsiella pneumoniae*	NDM
2612	*Klebsiella pneumoniae*	ESBL
2613	*Escherichia coli*	ESBL
2614	*Klebsiella pneumoniae*	ESBL
2615	*Klebsiella pneumoniae*	NDM
2616	*Escherichia coli*	ESBL

2617	*Escherichia coli*	ESBL
2618	*Escherichia coli*	ESBL
2619	*Escherichia coli*	ESBL
2620	*Escherichia coli*	ESBL
2621	*Klebsiella pneumoniae*	ESBL
2622	*Escherichia coli*	ESBL
2623	*Enterobacter cloacae complex*	VIM
2624	*Escherichia coli*	ESBL
2625	*Klebsiella pneumoniae*	ESBL
2626	*Staphylococcus aureus*	MRSA
2627	*Staphylococcus aureus*	MRSA
2628	*Staphylococcus aureus*	MRSA
2629	*Klebsiella pneumoniae*	ESBL
2630	*Escherichia coli*	ESBL
2631	*Escherichia coli*	OXA48
2632	*Klebsiella pneumoniae*	ESBL
2633	*Klebsiella pneumoniae*	ESBL
2634	*Staphylococcus aureus*	MRSA
2635	*Escherichia coli*	ESBL
2636	*Escherichia coli*	ESBL
2637	*Klebsiella oxytoca*	ESBL
2638	*Klebsiella pneumoniae*	ESBL
2639	*Klebsiella pneumoniae*	ESBL
2640	*Escherichia coli*	ESBL
2641	*Staphylococcus aureus*	MRSA
2642	*Klebsiella pneumoniae*	ESBL
2643	*Klebsiella pneumoniae*	ESBL
2644	*Staphylococcus aureus*	MRSA
2645	*Klebsiella pneumoniae*	ESBL
2646	*Enterobacter cloacae complex*	OXA48
2647	*Klebsiella pneumoniae*	ESBL
2648	*Klebsiella pneumoniae*	OXA48
2649	*Klebsiella pneumoniae*	ESBL
2650	*Escherichia coli*	ESBL
2651	*Enterobacter cloacae complex*	ESBL
2652	*Enterobacter cloacae complex*	ESBL
2653	*Escherichia coli*	ESBL
2654	*Escherichia coli*	ESBL
2655	*Klebsiella pneumoniae*	ESBL
2656	*Staphylococcus aureus*	MRSA
2657	*Escherichia coli*	ESBL
2658	*Escherichia coli*	ESBL
2659	*Escherichia coli*	ESBL
2660	*Escherichia coli*	ESBL
2661	*Escherichia coli*	ESBL
2662	*Escherichia coli*	ESBL

2663	*Escherichia coli*	ESBL
2664	*Escherichia coli*	ESBL
2665	*Citrobacter freundii*	ESBL
2666	*Klebsiella pneumoniae*	ESBL
2667	*Klebsiella pneumoniae*	ESBL
2668	*Citrobacter amalonaticus*	ESBL
2669	*Klebsiella variicola*	ESBL
2670	*Escherichia coli*	ESBL
2671	*Escherichia coli*	ESBL
2672	*Enterobacter cloacae complex*	ESBL
2673	*Enterobacter cloacae complex*	ESBL
2674	*Escherichia coli*	ESBL
2675	*Klebsiella pneumoniae*	ESBL
2676	*Klebsiella pneumoniae*	ESBL
2677	*Escherichia coli*	ESBL
2678	*Enterobacter cloacae complex*	ESBL
2679	*Escherichia coli*	ESBL
2680	*Klebsiella pneumoniae*	OXA48
2681	*Klebsiella pneumoniae*	ESBL
2682	*Staphylococcus aureus*	MRSA
2683	*Klebsiella pneumoniae*	ESBL
2684	*Staphylococcus aureus*	MRSA
2685	*Escherichia coli*	ESBL
2686	*Enterobacter cloacae complex*	ESBL
2687	*Klebsiella pneumoniae*	NDM, OXA48
2688	*Citrobacter freundii*	ESBL
2689	*Escherichia coli*	ESBL
2690	*Klebsiella pneumoniae*	ESBL
2691	*Klebsiella pneumoniae*	ESBL
2692	*Escherichia coli*	ESBL
2693	*Escherichia coli*	ESBL
2694	*Escherichia coli*	ESBL
2695	*Citrobacter freundii*	ESBL
2696	*Klebsiella pneumoniae*	ESBL
2697	*Klebsiella pneumoniae*	ESBL
2698	*Escherichia coli*	ESBL
2699	*Escherichia coli*	NDM
2700	*Escherichia coli*	ESBL
2701	*Escherichia coli*	OXA48
2702	*Escherichia coli*	ESBL
2703	*Klebsiella pneumoniae*	NDM, OXA48
2704	*Escherichia coli*	ESBL
2705	*Escherichia coli*	ESBL
2706	*Escherichia coli*	ESBL
2707	*Klebsiella pneumoniae*	ESBL

2708	*Escherichia coli*	ESBL
2709	*Escherichia coli*	ESBL
2710	*Staphylococcus aureus*	MRSA
2711	*Klebsiella pneumoniae*	ESBL
2712	*Escherichia coli*	ESBL
2713	*Escherichia coli*	ESBL
2714	*Escherichia coli*	ESBL
2715	*Klebsiella pneumoniae*	OXA48
2716	*Citrobacter freundii*	NDM
2717	*Klebsiella pneumoniae*	ESBL
2718	*Staphylococcus aureus*	MRSA
2719	*Escherichia coli*	ESBL
2720	*Staphylococcus aureus*	MRSA
2721	*Escherichia coli*	ESBL
2722	*Klebsiella pneumoniae*	ESBL
2723	*Escherichia coli*	ESBL
2724	*Escherichia coli*	ESBL
2725	*Escherichia coli*	ESBL
2726	*Klebsiella pneumoniae*	ESBL
2727	*Citrobacter koseri*	ESBL
2728	*Klebsiella pneumoniae*	ESBL
2729	*Escherichia coli*	ESBL
2730	*Escherichia coli*	ESBL
2731	*Escherichia coli*	ESBL
2732	*Escherichia coli*	ESBL
2733	*Citrobacter freundii*	ESBL
2734	*Escherichia coli*	ESBL
2735	*Klebsiella pneumoniae*	ESBL
2736	*Escherichia coli*	ESBL
2737	*Klebsiella pneumoniae*	ESBL
2738	*Enterobacter cloacae complex*	ESBL
2739	*Escherichia coli*	ESBL
2740	*Klebsiella pneumoniae*	ESBL
2741	*Staphylococcus aureus*	MRSA
2742	*Klebsiella pneumoniae*	ESBL
2743	*Escherichia coli*	ESBL
2744	*Klebsiella pneumoniae*	ESBL
2745	*Escherichia coli*	NDM
2746	*Escherichia coli*	ESBL
2747	*Acinetobacter baumannii*	OXA23
2748	*Klebsiella pneumoniae*	ESBL
2749	*Escherichia coli*	ESBL
2750	*Escherichia coli*	ESBL
2751	*Escherichia coli*	ESBL
2752	*Escherichia coli*	ESBL
2753	*Citrobacter freundii*	ESBL

2754	*Klebsiella pneumoniae*	NDM
2755	*Klebsiella pneumoniae*	OXA48
2756	*Escherichia coli*	ESBL
2757	*Escherichia coli*	ESBL
2758	*Escherichia coli*	ESBL
2759	*Escherichia coli*	ESBL
2760	*Escherichia coli*	ESBL
2761	*Escherichia coli*	ESBL
2762	*Escherichia coli*	OXA48
2763	*Escherichia coli*	ESBL
2764	*Escherichia coli*	ESBL
2765	*Escherichia coli*	ESBL
2766	*Enterobacter cloacae complex*	ESBL
2767	*Klebsiella pneumoniae*	ESBL
2768	*Escherichia coli*	ESBL
2769	*Escherichia coli*	ESBL
2770	*Escherichia coli*	ESBL
2771	*Escherichia coli*	ESBL
2772	*Klebsiella pneumoniae*	ESBL
2773	*Klebsiella pneumoniae*	ESBL
2774	*Klebsiella pneumoniae*	ESBL
2775	*Escherichia coli*	ESBL
2776	*Citrobacter freundii*	OXA48
2777	*Escherichia coli*	ESBL
2778	*Klebsiella pneumoniae*	ESBL
2779	*Escherichia coli*	ESBL
2780	*Citrobacter freundii*	ESBL
2781	*Klebsiella pneumoniae*	ESBL
2782	*Escherichia coli*	ESBL
2783	*Staphylococcus aureus*	MRSA
2784	*Escherichia coli*	ESBL
2785	*Klebsiella pneumoniae*	ESBL
2786	*Klebsiella pneumoniae*	ESBL
2787	*Escherichia coli*	ESBL
2788	*Escherichia coli*	ESBL
2789	*Klebsiella pneumoniae*	ESBL
2790	*Staphylococcus aureus*	MRSA
2791	*Escherichia coli*	ESBL
2792	*Escherichia coli*	ESBL
2793	*Escherichia coli*	ESBL
2794	*Escherichia coli*	ESBL
2795	*Klebsiella pneumoniae*	ESBL
2796	*Staphylococcus aureus*	MRSA
2797	*Escherichia coli*	ESBL
2798	*Klebsiella pneumoniae*	ESBL
2799	*Escherichia coli*	ESBL

2800	*Escherichia coli*	ESBL
2801	*Klebsiella pneumoniae*	ESBL
2802	*Klebsiella pneumoniae*	ESBL
2803	*Klebsiella pneumoniae*	ESBL
2804	*Escherichia coli*	OXA48
2805	*Enterobacter cloacae complex*	ESBL
2806	*Escherichia coli*	ESBL
2807	*Escherichia coli*	ESBL
2808	*Escherichia coli*	ESBL
2809	*Escherichia coli*	ESBL
2810	*Staphylococcus aureus*	MRSA
2811	*Escherichia coli*	ESBL
2812	*Escherichia coli*	ESBL
2813	*Enterobacter cloacae complex*	ESBL
2814	*Escherichia coli*	ESBL
2815	*Escherichia coli*	ESBL
2816	*Klebsiella pneumoniae*	ESBL
2817	*Escherichia coli*	ESBL
2818	*Staphylococcus aureus*	MRSA
2819	*Raoultella ornithinolytica*	ESBL
2820	*Klebsiella pneumoniae*	OXA48
2821	*Escherichia coli*	ESBL
2822	*Escherichia coli*	ESBL
2823	*Escherichia coli*	ESBL
2824	*Enterobacter cloacae complex*	ESBL
2825	*Escherichia coli*	ESBL
2826	*Escherichia coli*	ESBL
2827	*Klebsiella pneumoniae*	ESBL
2828	*Klebsiella oxytoca*	NDM, OXA48
2829	*Escherichia coli*	ESBL
2830	*Klebsiella pneumoniae*	NDM
2831	*Escherichia coli*	ESBL
2832	*Klebsiella pneumoniae*	ESBL
2833	*Escherichia coli*	ESBL
2834	*Klebsiella pneumoniae*	ESBL
2835	*Escherichia coli*	ESBL
2836	*Escherichia coli*	ESBL
2837	*Citrobacter freundii*	ESBL
2838	*Staphylococcus aureus*	MRSA
2839	*Acinetobacter baumannii*	OXA23
2840	*Klebsiella pneumoniae*	ESBL
2841	*Escherichia coli*	ESBL
2842	*Klebsiella pneumoniae*	ESBL
